# Quantum Tunnelling Effects in the Guanine-Thymine Wobble Misincorporation via Tautomerism

**DOI:** 10.1021/acs.jpclett.2c03171

**Published:** 2022-12-23

**Authors:** Louie Slocombe, Max Winokan, Jim Al-Khalili, Marco Sacchi

**Affiliations:** † Leverhulme Quantum Biology Doctoral Training Centre, University of Surrey, GuildfordGU2 7XH, U.K.; ‡ Department of Physics, University of Surrey, GuildfordGU2 7XH, U.K.; § Department of Chemistry, University of Surrey, GuildfordGU2 7XH, U.K.

## Abstract

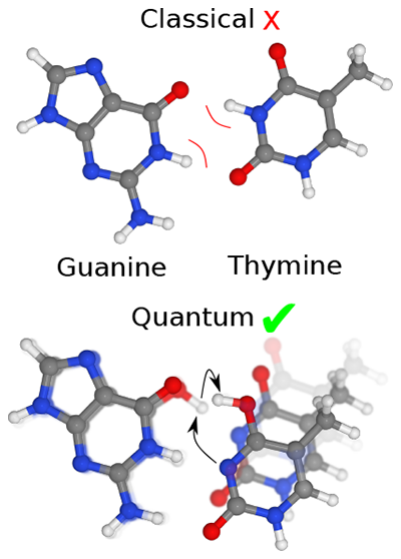

The misincorporation of a noncomplementary DNA base in the polymerase active site is a critical source of replication errors that can lead to genetic mutations. In this work, we model the mechanism of wobble mispairing and the subsequent rate of misincorporation errors by coupling first-principles quantum chemistry calculations to an open quantum systems master equation. This methodology allows us to accurately calculate the proton transfer between bases, allowing the misincorporation and formation of mutagenic tautomeric forms of DNA bases. Our calculated rates of genetic error formation are in excellent agreement with experimental observations in DNA. Furthermore, our quantum mechanics/molecular mechanics model predicts the existence of a short-lived “tunnelling-ready” configuration along the wobble reaction pathway in the polymerase active site, dramatically increasing the rate of proton transfer by a hundredfold, demonstrating that quantum tunnelling plays a critical role in determining the transcription error frequency of the polymerase.

DNA polymerase is an enzyme that catalyzes the synthesis of DNA molecules by matching complementary deoxyribonucleoside triphosphates (dNTP) to the template DNA strand using the standard Watson–Crick (WC) base pair rules. However, when a noncomplementary dNTP diffuses into the active site during the polymerase dNTP sampling, the polymerase domain will undergo a transition from an open to an ajar conformation, thus forming a different nonstandard hydrogen-bonded base-pairing arrangement called wobble mispair. While there are other sources of replication errors, the fidelity of replication primarily depends on the ability of polymerases to select and incorporate the correct complementary base (see Figure 1) and reject wobble mispairs. However, it is proposed^1−8^ that a wobble mismatch can form alternative tautomeric configurations that can mimic the WC geometry and lead to erroneous DNA base matches, such as wobble(G-T) → G*-T, where G* is the tautomeric (enol) form of the G base.^9^ Watson–Crick-like mispairs have been observed in the active sites of DNA polymerases^1,10,11^ and ribosomes in enzymatically competent conformations.^2,12,13^ Both nuclear magnetic resonance (NMR) relaxation dispersion experiments and simulations^3−5^ indicate that the concentration of tautomeric mismatches in the cellular environment is significant and has a considerable impact on the replication fidelity of the polymerase. Furthermore, the previous work demonstrates that the population of Watson–Crick-like G-T mispairs depends on the local environment, such as the base sequence and the local solvation environment.

**Figure 1:**
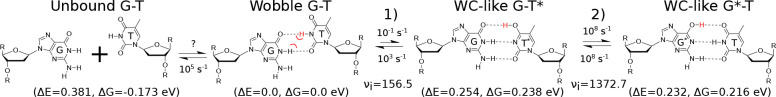
Schematic representation of the G-T wobble mispair and the conversion to a Watson–Crick-like configuration via a proton transfer process. For reaction 1 to occur and for the wobble confirmation to adopt a Watson–Crick-like configuration, a proton must rearrange (red). The reaction rates are shown above the arrows. Reaction 1 competes with the unbinding rate of the wobble mispair shown by the first set of arrows on the left. Reaction 2 denotes the further proton transfer reaction in the Watson–Crick-like configuration. Here, the asterisk denotes the tautomeric enol form of the base.

Recent theoretical work on the Watson–Crick bonded bases entering the helicase enzyme has shown that quantum effects lead to the formation of metastable tautomeric forms of DNA.^14,15^ Quantum chemical models of G-C and A-T base pairs^14^ describe the double proton transfer’s potential energy surface (PES) in both canonical base pairs. The main difference between the A-T and G-C PES is that A-T has a considerable forward barrier for tautomer formation but a small reverse barrier that causes its tautomeric form to be unstable.^16−18^ On the contrary, G-C has a sizable reverse barrier, giving a tautomeric lifetime comparable to that of the replication process. Moreover, quantum tunnelling leads to a fast proton exchange between the bases,^19^ such that the time scale of the helicase cleavage is much slower than the proton transfer dynamics.^9^ Consequently, using a semiclassical interpretation,^14,15^ the potentially mutagenic tautomer is continuously formed and destroyed over time scales several orders of magnitude quicker than that of helicase cleavage, after which, the bases are split into their monomeric forms. However, using a quantum interpretation, the tunnelling proton’s wave function evolve on a shorter time scale, so two probability distributions (in the canonical and tautomeric configuration) emerge. As previously demonstrated, once the tautomer is formed and the DNA is opened, it is stabilized and unlikely to revert to its canonical form due to a prohibitively large reaction barrier.^14,20^ However, the degree to which environmental effects play a role in destabilizing the tautomer still needs to be determined. Some initial evidence suggests that the DNA^17^ environment reduces the reverse barrier, but it is unclear for the DNA and helicase complex.

Recent NMR experiments using isotopic substitution suggest that when the DNA enters the polymerase active site, the wobble tautomerization reaction might be facilitated by tunnelling.^21^ Rangadurai et al. investigated the dynamics of the transition between a wobble and Watson–Crick-like G-T in duplex DNA by performing NMR relaxation dispersion in both H_2_O and D_2_O. The authors reported that the kinetic isotope effect (KIE) in the exchange rate between the two conformations of the mismatch was 3-fold slower in heavy water. This result provides the first experimental evidence supporting the hypothesis that quantum effects are involved in wobble tautomerization.

In the replication machinery, during the polymerase dNTP sampling, the sample is rejected if G is mismatched against T. We propose a reaction pathway that connects the wobble mismatch to a Watson–Crick-like pairing (shown as pathway 1 in Figure 1), leading to base misincorporation through a phosphodiester bond formation. In this scheme, proton transfer must occur for the bases to slide to a Watson–Crick-like pairing, either classically, via an “over the barrier” mechanism, or via quantum tunnelling. To avoid replication errors, the polymerase must reject such mismatches; otherwise, the wrong base pairing can undergo further proton transfer, connecting two Watson–Crick-like tautomeric forms (shown as pathway 2). Additional pathways are explored in Supplementary Note 1.

We investigate the reactions wobble(G-T) *⇌* G-T* (reaction 1) and wobble(G-T) *⇌* G*-T, whereby the reactants start as a wobble mismatch and, via proton transfer, result in a Watson–Crick-like conformation. We determine that the reaction wobble(G-T) to G*-T proceeds through a G-T* intermediate state in a stepwise mechanism. The minimum energy pathway can therefore be described by two steps; in the first step, the wobble(G-T) passes through the transition state of wobble(G-T) *⇌* G-T*. In the second step, through an intermediate local minimum, the G-T* intermediate converts to G*-T.^6,22^ In comparison, the G-T* reaction (reaction 1) contains one transition state with no intermediate minimum.

Figure 2 shows the minimum energy path of this reaction, for which the forward barrier is 0.926 eV and the reverse barrier is 0.680 eV. We perform a normal-mode analysis to calculate the free energy values of the reactant, transition state, and product. We determine that the free energy values are smaller than the electronic energy barriers. The free energy contributions decrease the forward barrier by 20% and the reverse barrier by 30%, resulting in a free energy profile consistent with the work of Li et al.^5^ A summary can be found in Supplementary Note 1, and a detailed comparison of the reaction barrier parameters to the literature in Supplementary Note 3.

**Figure 2:**
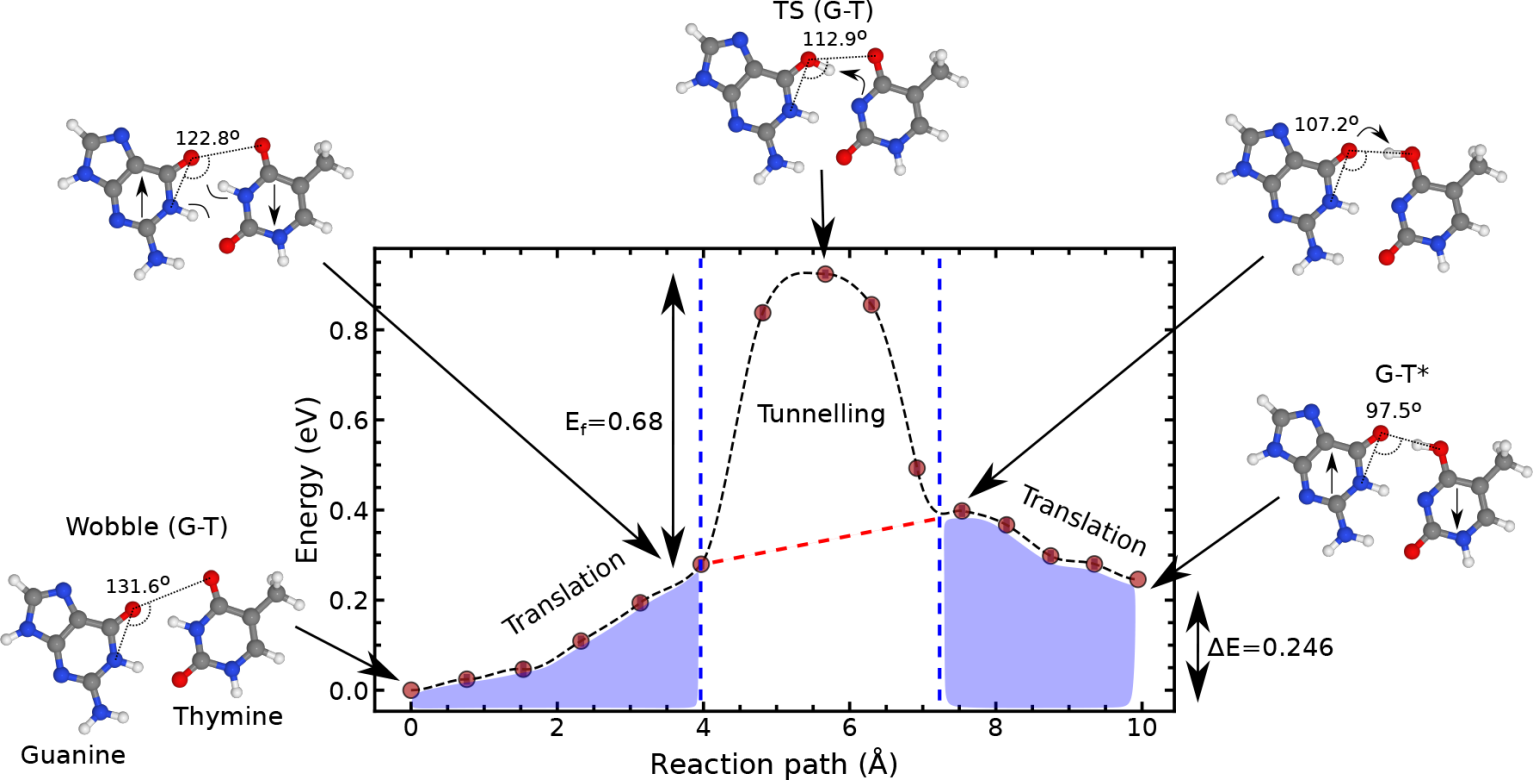
Minimum energy path of the wobble(G-T) *⇌* G-T* proton transfer reaction pathway. The reaction 1 paths are obtained using a machine learning approach to the nudged elastic band method. The reaction path contains classical rearrangement of the bases and a high reaction barrier through which the proton can tunnel.

On the wobble(G-T) *⇌* G-T* reaction path (reaction 1), we observe three regions (see Figure 2). The first region (0–4 Å) largely corresponds to the collective movement of the bases relative to each other as they drift to a Watson–Crick-like bonding angle. In this region, the Δ*E* is essentially constant as the molecules move over a flat PES in which weak van der Waals interactions dominate. The fast and activated proton transfer occurs between 4 and 7.5 Å. In this region, the proton of the thymine N–H bond first transfers to the oxygen of the carboxylic group of G (as described by the arrow in the transition state of Figure 2). The same proton subsequently hops back to the nearest oxygen of T. Finally, the region of the reaction path closest to the product (>7.5 Å) corresponds to a further collective translation of the bases toward a Watson–Crick-like configuration, with little rearrangement in the bond of the transferred proton.

Despite several previous attempts to model the creation of G-T wobble mismatches,^5,6,22^ the presence and role of quantum effects in this reaction have not been addressed, with previously reported semiclassical models severely underestimating the experimental reaction rates. In the following, we introduce a first-principles-based quantum dynamic approach for modeling proton tunnelling in a realistic cellular environment, which accounts for the noise and thermal fluctuations of the biological system. We then employ this method to calculate the G-T wobble mismatch reaction pathway to the Watson–Crick-like configuration and the double proton transfer scheme in the Watson–Crick-like configuration (see Figure 1). Quantum and classical contributions to the reaction rate are determined, and we discuss the contribution of proton tunnelling in forming Watson–Crick-like tautomers within the polymerase active site.

The open quantum systems approach employed in this study is based on Caldeira and Leggett’s quantum Brownian motion model^23^ in which the protons in the hydrogen bonds are embedded in an ohmic bath of quantum oscillators, which represent the cellular environment. The interactions between the DNA and the environment are integrated over time using the path integral formalism introduced by Feynman and Vernon.^24^ The equivalent phase-space version is given by the Wigner-Moyal Caldeira and Leggett equation (WM-CL)
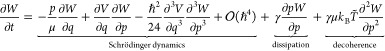
1where *W* is a quasi-probability density encapsulating the proton’s quantum state as a function of both position (*q*) and momentum (*p*).^25,26^ The first set of terms in eq 1 corresponds to the Schrödinger dynamics of a particle with effective mass μ. The subsequent two terms correspond to the dissipation and decoherence arising from the coupling to the quantum bath. γ is the phenomenological friction constant that describes the strength of the coupling to the bath;^23^*k*_B_ is Boltzmann’s constant, and *T̃* represents the effective bath temperature.

The advantage of employing an open quantum systems model is that it incorporates the interactions with the local environment in the quantum dynamics. These interactions significantly affect the system’s dynamics and can either impede or encourage the system’s evolution, known as a quantum Zeno or anti-Zeno effect.^27^ Furthermore, the coupling to the environment results in quantum dissipation, such that the information in the system is lost to its environment and decoherence, where a quantum system loses its wave-like properties. As a consequence, classical behavior emerges.

Assuming that the system-to-environment coupling constant is dominated by the thermal fluctuations of the surrounding water molecules, we can estimate the value of γ. Water has a vibrational spectrum in the range of 3300–3900 cm^–1^.^28^ Therefore, assuming that the fastest oscillators in this range dominate, we use an ohmic spectral density for our environment oscillators^23^ having a coupling parameter γ of 3900 cm^–1^. We determine the quantum contribution to the reaction rate by monitoring the flux of the density passing through the transition state (see Supplementary Note 2 for further details). The forward and reverse reaction rate constants, *k*_f_ and *k*_r_, respectively, are obtained from
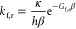
2where β = 1/(*k*_B_*T*) and *G*_f_ and *G*_r_ correspond to the Gibbs free energy barrier of the forward and reverse reactions, respectively. The tunnelling factor, *κ*, encapsulates the quantum-to-classical contribution to the rate, incorporating quantum effects such as tunnelling and nonclassical reflections.

First, we determine the quantum and classical rates for reaction 1 using our open quantum systems approach. Reaction 1 has a prohibitively high and wide reaction barrier (see Figure 2), resulting in a low classical and quantum reaction rate. We evaluate that the quantum-to-classical ratio is small (*κ* = 1.02), suggesting that tunnelling is negligible; here, dissipative and decoherent effects from the biological environment suppress the tunnelling. We find that the overall reaction rate is dominated by an over-the-barrier classical mechanism, with a value of 5.244 × 10^–1^ s^–1^, which is consistent with the experimental value (0.6–68 s^–1^)^4,21^ of the G-T wobble system in DNA. The reaction rate is several orders of magnitude smaller than the dNTP unbinding rate, which is on the order of 70 000 s^–1^.^4^ Furthermore, we determine the effect of isotopic substitutions on the reaction rate and find that the reaction rate is essentially unaffected by deuterium substitution (KIE = 1.1). Consequently, due to the slow reaction rate, the dNTP unbinding rate and subsequent base rejection compete with the proton transfer mechanism. As a result, statistically, some of the wobble mismatches will eventually diffuse from the polymerase’s active site before proton transfer occurs. Because the diffusion time scale competes with the proton transfer time scale, the final population of tautomers incorporated will be reduced as described by the kinetic network in ref (4).

To compare how the change in environment and the subsequent change to the reaction profile impact the tunnelling, we extract the free energy pathway data from ref (5) and apply our tunnelling approach. A detailed description can be found in Supplementary Note 3. In summary, we determine that regardless of whether the G-T wobble is exclusively in an aqueous solution or a more complex DNA environment, the tunnelling is primarily suppressed to the degree that it is insignificant.

However, we note that the PES in Figure 2 describes three fundamentally different molecular motions, and only the inner barrier (section 2 in Figure 2) corresponds to the proton transfer between the bases. In contrast, regions 1 and 3 correspond to overall translations of the bases without significant changes in the hydrogen bond length. This PES topology is compatible with a tunnelling-ready state composed of the reactant’s activated structure seen at the end of region 1. The activation process concerns the reorganization of the non-hydrogen atoms where thermal energy is required for the reactants to reach an activated tunnelling-ready state, whereby the reactant and product states become similar in energy.

Here we explore the minimum energy pathway of proton transfer in the tunnelling-ready state. Further details of the methods can be found in Supplementary Note 1. The subsequent minimum energy pathway is shown in Figure 3. Here, the reaction pathway shows three minima corresponding to the bases already partially slid into a Watson–Crick-like shape, the second where the proton has transferred to the other base, and the third the return of the proton back to the same base. The last two minima indicate that if we assume that the proton transfer is much faster than the rest of the atomic motion during the reaction, a bifurcation of the reaction pathway is possible. In fact, after the first initial proton transfer, the rest of the atoms could rearrange, trapping the population in the middle well.

**Figure 3:**
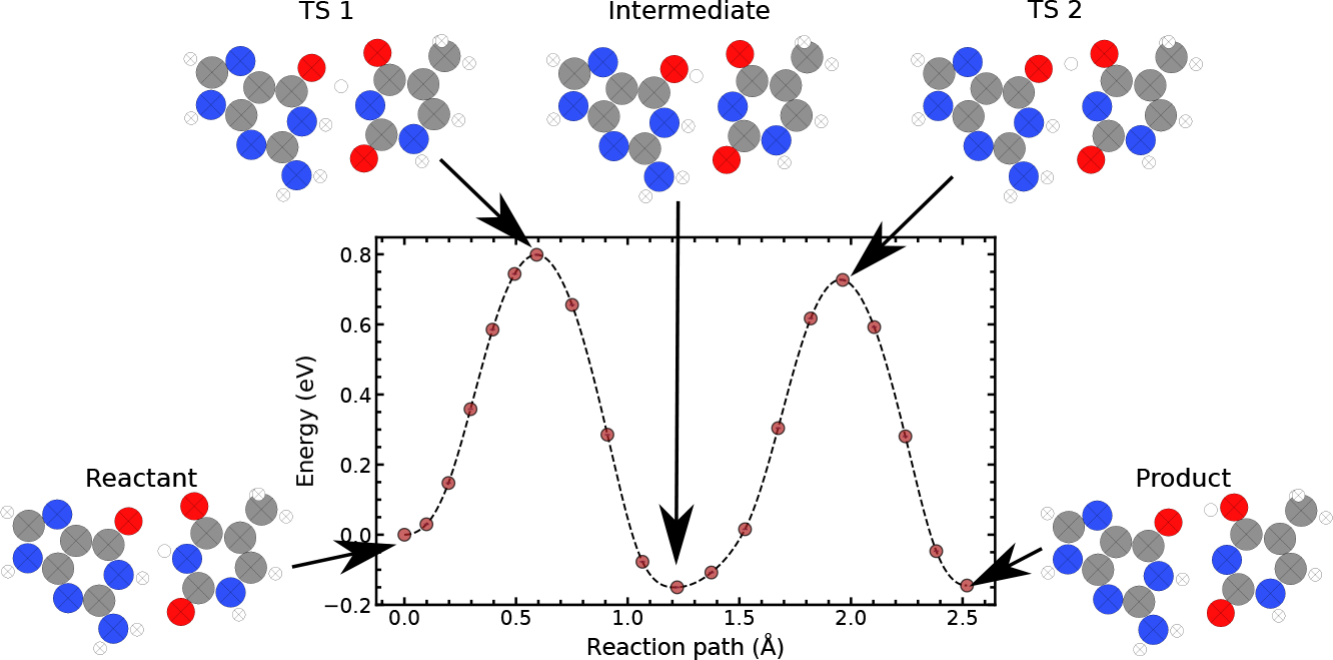
Tunnelling-ready minimum energy path of the wobble(G-T) ⇌ G-T* reaction. The proton transfer reaction pathway, reaction 1, assumes that the bases have already partly slid into a Watson–Crick-like shape. Each minimum and each maximum along the path are labeled. Crossed-out atoms indicate that they have been constrained.

To calculate the contribution of quantum tunnelling in this activated tunnelling-ready state, we re-evaluate the PES considering only where the proton is transferring. We then calculate the rate of proton tunnelling from the tunnelling-ready state through region 2. We calculate the inner barrier section (between 4 and 7.5 Å) by taking the image of the start of the barrier from reaction 1 and assuming that the local polymerase environment has thermally induced this conformational change. Using this approach, we calculate the quantum contribution to the reaction rate and find that the overall rate is much larger. The rate is now 1.279 × 10^–1^ s^–1^, with a *κ* of 99.0 indicating a large contribution from tunnelling. By substituting hydrogen with deuterium, we find that the tunnelling-corrected reaction rate exhibits a KIE of 10.15, which is compatible with experimental results^4^ that predict a 3-fold decrease in rate.

We now focus on explicitly how the polymerase active site interacts with the G-T wobble mismatch and the tunnelling-ready state. Free energy pathway calculations by Li et al.^5^ suggest that the polymerase introduces a 46% increase in the proton transfer reaction barrier. However, as the density functional theory calculations show, for the wobble(G-T) ⇌ G-T* reaction to occur, the nucleotide dimer must first be compressed into a tunnelling-ready state.^29^ Therefore, it is desirable to know whether this state is populated in a biologically relevant thermal ensemble. To this end, hybrid quantum classical quantum-mechanical/molecular-mechanical molecular dynamics (QM/MM MD) simulations are performed, wherein the entire polymerase enzyme and solvent are included explicitly in the simulation system. Several short replica simulations are computed from the wobble configuration obtained through a crystal structure, totalling >2800 ps of QM/MM MD. This investigation is repeated without the enzyme to highlight the compressing effect of the enzyme’s “thumb” region. Both simulation systems are described and illustrated in Supplementary Note 4.

A metric for the overlap distance between the simulation snapshot and the tunnelling-ready state from the ML-NEB calculations is defined as Δ. Delta measures how often the tunnelling-ready state is populated in a biologically relevant thermal ensemble to be determined. Using these QM/MM MD simulations, a cumulative histogram of this data is shown in Figure 4 for both aqueous DNA and the polymerase DNA complex. Full computational details are provided in Supplementary Note 4.

**Figure 4:**
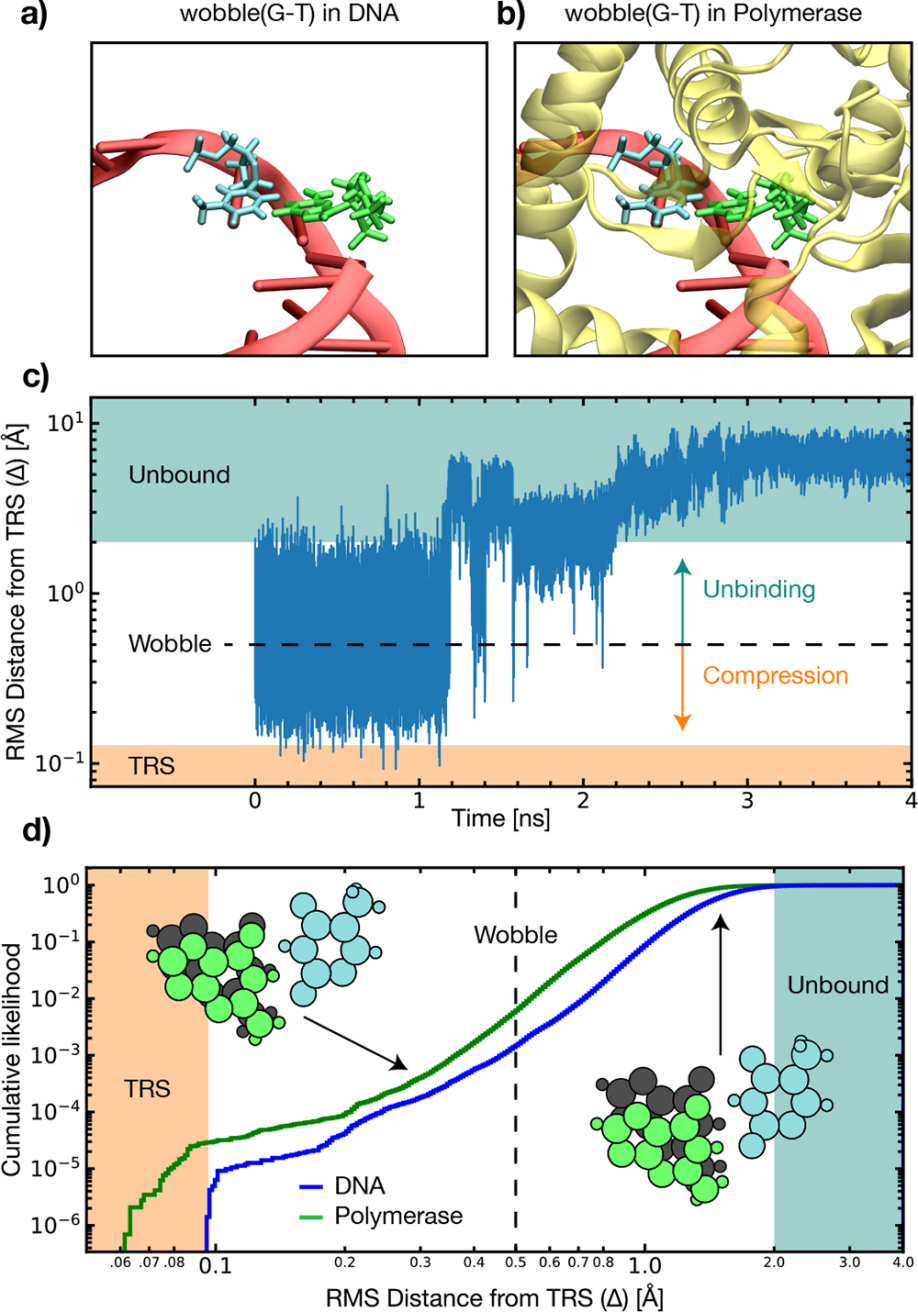
Dynamical investigation of the biological relevance of the compressed tunnelling-ready state (TRS) of the wobble(G-T) mismatch. The compression of the wobble(G-T) mismatch is considered in a DNA insertion site with the polymerase enzyme (b) and without the enzyme (a). An RMS distance is defined to the TRS and plotted (c) during a single long molecular dynamics trajectory and (d) aggregated from >2800 ps of QM/MM MD simulations. In panel c, the RMS distance to the wobble(G-T) configuration is shown as a black dashed line and two additional regimes are illustrated. First, an unbound regime is defined with a Δ of >2.0 Å, and a set of tunnelling-ready/compressed states with a Δ of <0.096 Å. In panel d, the cumulative likelihood across a range of Δ values is plotted for the polymerase–DNA complex (green line) and aqueous DNA (blue line). In this context, the cumulative likelihood determines the probability of finding the dimer at an RMS distance below the given value. Two example conformations are shown relative to the TRS (gray circles) position.

The MD simulations demonstrate that the G-T dimer either remains in a wobble configuration consistent with the reactant configuration or exists in a transitory unbound state (see the schematic representation in Figure 1). Among all of the QM/MM MD replica simulations, 0.003% of the trajectory is within 0.096 Å root-mean-square distance of the tunnelling-ready state for the enzyme–DNA complex. Without the enzyme, no Δ values of <0.096 are observed. The lower Δ regime corresponding to the TRS is informed by considering the Δ difference between the fifth and sixth ML-NEB data points from Figure 2. Assuming a uniform distribution of events, this is equivalent to the dimer compressing once every 35.2 ps in the polymerase active site. Despite the approximately 0.275 eV energetic penalty to compression shown in Figure 2, our dynamical simulations show that this state can be reached within a realistic biological environment. Crucially, in the absence of the enzyme, no population is found below a Δ of 0.97 Å, suggesting that the enzyme facilitates the compression. These results justify performing proton transfer calculations for the wobble(G-T) ⇌ G-T* reaction from such a compressed tunnelling-ready state as shown in Figure 3. While the proton transfer mechanism starts from a more compressed G-T wobble conformation, reaction rate calculations must now also consider the sparsity with which this compressed state is observed, as the barrier shown in Figure 3 is accessible only <0.003% of the time.

On the contrary, for the G*-T *⇌* G-T* reaction (reaction 2 in Figure 1), the barrier is considerably smaller than for the wobble transfer reaction (reaction 1 in Figure 1), 0.356 eV versus 0.926 eV. As shown in Figure 5, the region of the PES comprised between 0.0 and 0.5 Å corresponds to the small translation of the two bases toward each other to facilitate the transfer. First, the middle proton in the N–H–N bond transfers (denoted by the arrow in Figure 5). Then, the O–H–O proton transfers, as evidenced by the presence of a shoulder in the PES after the transition state.

**Figure 5:**
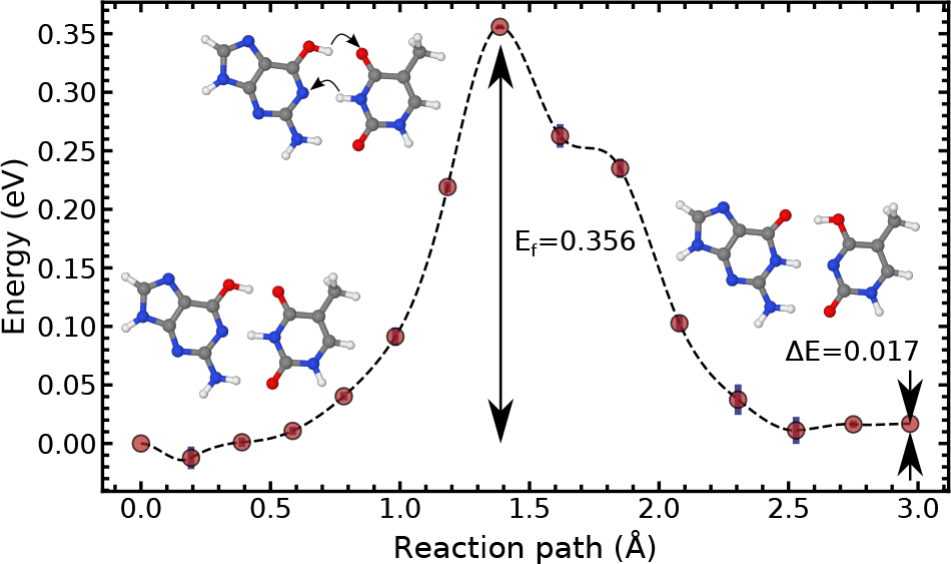
Minimum energy path of the G*-T *⇌* G-T* reaction. The double proton transfer reaction pathway, reaction 2, assuming the conversion to a Watson–Crick-like state has already occurred.

Here, the forward reaction barrier (*E*_f_ = 0.356 eV) and asymmetry (Δ*E* = 0.017 eV) are small compared to those of the wobble-to-enol transfer and compare well with previous calculations (*E*_f_ = 0.34 eV^5^). The low reaction barrier leads to a fast proton transfer with a large forward and reverse reaction rate on the order of 10^8^ s^–1^. We use the open quantum systems model to determine a quantum-to-classical rate ratio *κ* of 18.1 and a KIE of 4.25. The high *κ* and KIE for this reaction suggest that quantum effects play a significant role in reaction 2 and that, due to the fast proton transfer time scale, the system can quickly reach equilibrium. After a single proton transfer successfully forms the tautomeric Watson–Crick-like form, the protons can continue to transfer between the bases (reaction 2) via a fast double proton transfer. Consequently, following another step of replication in the polymerase, an error will likely be induced on both daughter strands, as the enol forms will readily mismatch with the wrong base.^16^

In summary, we have employed quantum chemical calculations to determine the reaction pathway of several reactions for generating tautomers of the G-T wobble mispair. We applied an open quantum systems approach to account for the decoherent and dissipative local environment^23^ and identified quantum and classical contributions to the reaction rates. For the wobble(G-T) *⇌* G*-T mechanism, we found that the reaction proceeds via a stepwise process involving G-T*. Consequently, we focused on the wobble(G-T) *⇌* G-T* reaction. The proton transfer reaction from the wobble to the Watson–Crick pathway has a significantly high and broad reaction barrier, which implies an insignificant contribution from quantum tunnelling and a slow classical rate. We noted that for the wobble(G-T) ⇌ G-T* reaction to occur, the nucleotide dimer must first be compressed into a tunnelling-ready state; we probed this state using QM/MM MD to determine how likely it is populated in a biologically relevant thermal ensemble. We determined that this state is more likely to be populated in the polymerase environment and leads to an increase in the level of quantum tunnelling.

As highlighted by previous computational studies, the role of proton transfer in spontaneous mutation is a complex affair.^5,8,17^ However, the proton transfer mechanism in the polymerase is a prominent candidate as a source of mutations as it is later in the replication cycle and could play a role that is more significant than that played by other equilibria competing during mutation. Furthermore, for mechanisms involving double-stranded Watson–Crick DNA, it needs to be clarified if the helicase or another mechanism reduces the proton transfer populations via electrostatic destabilization or exonuclease proofreading mechanisms.

To conclude, our model predicts tunnelling rates that match the experimental NMR observed rates to a high degree of accuracy, opening the possibility that quantum mechanics is required to explain the biologically relevant functionality of polymerase.

## Data Availability

The data for the reaction pathway are available on Github. Additionally, data presented in this article are available from the corresponding authors upon reasonable request.

## Supporting Information Available

A detailed description of the methods and theory used to generate potential energy reaction surfaces and the parameters used to model the tunnelling (PDF)
Transparent Peer Review report available (PDF)
