# Supplementary material for: Quantum Tunnelling Effects in the Guanine-Thymine Wobble Misincorporation via Tautomerism
Source: J Phys Chem Lett. 2022 Dec 23;14(1):9–15. doi: 10.1021/acs.jpclett.2c03171 (PMC9841559; doi:10.1021/acs.jpclett.2c03171)
Supplement: Supplementary file 1 — jz2c03171_si_001.pdf [file jz2c03171_si_001.pdf]

Supporting Information: Quantum Tunnelling Effects in the Guanine-Thymine Wobble Misincorporation via Tautomerisation

Louie Slocombe*
Leverhulme Quantum Biology Doctoral Training Centre,
University of Surrey, Guildford, GU2 7XH, UK. and
Department of Chemistry, University of Surrey, Guildford, GU2 7XH, UK.

Max Winokan†
Leverhulme Quantum Biology Doctoral Training Centre,
University of Surrey, Guildford, GU2 7XH, UK.

Jim Al-Khalili‡
Department of Physics, University of Surrey, Guildford, GU2 7XH, UK.

Marco Sacchi§
Department of Chemistry, University of Surrey, Guildford, GU2 7XH, UK.
(Dated: December 16, 2022)

This document contains supplemental information for the results presented in the manuscript *Quantum Tunnelling Effects in the Guanine-Thymine Wobble Misincorporation via Tautomerisation.* In this file, unless otherwise stated, we use the Hartree atomic unit system, energy is in units of Hartrees E_h, the reduced Planck constant is $\hbar = 1$, and lengths are in Bohr radius a_0.

CONTENTS

Supplementary Note 1: Density Functional Theory Methods 2
Obtaining the Reaction Pathway 2
Obtaining an Effective Mass of the System 2
Double-Well Potential Energy Surface Representations 2
Tunnelling-Ready State: Assuming the Frozen Approximation 4
Summary of the Proton Transfer Energy Landscapes 5

Supplementary Note 2: Using Open Quantum Systems to Describe Tunnelling 10
The Wigner-Moyal Caldeira-Leggett Model 10
Low-temperature Correction 11
Numerically Solving 12
Obtaining a Quantum Corrected Reaction Rate 12
Kinetic Isotope Effect 13
Summary of the Quantum Tunnelling Effects in Proton Transfer 14

Supplementary Note 3: Comparing the Effect of the Environment 15
Extracting the Free Energy Pathway 15
Comparing the Free Energy Pathway 15
Comparing Environmental Effects on the Quantum Tunnelling 15

Supplementary Note 4: QM/MM Calculations 18
Ensemble Molecular Dynamics 18
Ensemble QM/MM MD 18
Compression reaction coordinate definition 19

Supplementary References 21

* louie.slocombe@surrey.ac.uk
† m.winokan@surrey.ac.uk
‡ J.Al-Khalili@surrey.ac.uk
§ m.sacchi@surrey.ac.uk

SUPPLEMENTARY NOTE 1: DENSITY FUNCTIONAL THEORY METHODS

Obtaining the Reaction Pathway

We performed Density Functional Theory (DFT) calculations with NWChem 7.0.2 [1] at the B3LYP+D3/6-311++G** level of theory. We use the B3LYP exchange-correlation functional [2] with Grimme DFT-D3 dispersion corrections to capture empirical long-range contributions [3, 4]. Based on previous works, this combination of exchange-correlation functional and basis set offers comparable accuracy to Møller–Plesset perturbation theory of the second order while at a fraction of the computing cost [5]. We embed the DNA bases in an implicit continuum solvation model [6–8] describing the influence of the surrounding water molecules, where $\epsilon = 78.4$.

We obtained the potential energy landscapes describing the proton transfer reactions using a machine learning approach to the classical all-nudged elastic band algorithm (ML-NEB) [9, 10]. The ML-NEB approach minimises the number of DFT single-point energy calculations required to depict the minimum energy path (MEP) accurately. In our treatment, we collect the movement of the protons transferring (and other atoms moving to facilitate the transfer) into a single axis. The reaction pathway contains a general description of the transfer process; the energetic landscape of this pathway is then explored using ML-NEB. The ML-NEB algorithm incorporates a Gaussian regression model to produce a surrogate description of the accurate MEP. Thus the uncertainty in the energy points on surrogate MEP becomes the convergence criteria.

The atomic simulation environment (ASE) [11, 12] was used throughout this work to connect NWChem to Python3 and the ML-NEB algorithm. All the structures were optimised using a force tolerance of $0.01\,\mathrm{eV\,\AA}^{-1}$. We adopt the optimised monomeric forms from Ref. [13], which are combined with their target base to form the hydrogen-bonded pair. We introduce free energy contributions by conducting a vibrational analysis at three points along the reaction coordinate: reactant, TS, and product structures; we use the ideal-gas limit to account for the translational and rotational degrees of freedom.

Obtaining an Effective Mass of the System

During the potential energy surface calculations, all degrees of freedom can relax. Consequently, the reaction pathway contains the joint motion of several atoms to facilitate the proton transfer reaction. Using the minimum energy pathway, we construct a reaction coordinate linking the reactant to the product via a transition state. We project the motion of each atom on to reaction coordinate.

To account for the contribution of the masses of the atoms participating in this reaction coordinate, we determine the effective mass μ using [14–18]

$$\mu = \sum_{i=1}^{N} m_i \left(\frac{\partial r_i}{\partial q} \cdot \frac{\partial r_i}{\partial q} \right). \tag{1}$$

Here the total number of atoms N, and m_i denotes the mass of the atom with index i, $m_i = (m_1, m_1, m_1, m_2, \ldots)$ 3N-sized vector of atomic masses. Whereas r_i is the 3N-sized Cartesian vector describing the change in the coordinates of atoms, with the reaction coordinate defined as q. The partial derivative of the Cartesian vector tracks the motion of atom m_i along the reaction coordinate q. The dot product normalises the motion relative to the collective rearrangement of all atoms. If there is no motion of atom i, it does not contribute to the reaction path.

Conversely, when atom i transfers, the dot product becomes non-trivial. Then by summing over all atoms i, we can obtain an effective mass which contains the contribution from all degrees of freedom. To evaluate the derivatives, we first pass the Cartesian coordinate vectors into a Savitzky–Golay filter to suppress any spurious noise introduced by the uncertainty in the path, which is inherent to the machine-learning approach to finding the reaction path. The filtered Cartesian coordinate vector and the reaction path are then interpolated using cubic splines.

Consequently, we determine the effective mass to account for the contribution of the masses of the atoms participating in this reaction coordinate. The result is shown in Fig. 1.

Double-Well Potential Energy Surface Representations

In the open quantum systems approach, we describe the proton transfer reactions using a pseudo-one-dimensional reaction coordinate connecting the reactant and product via a transition state barrier. To adopt the reaction profile

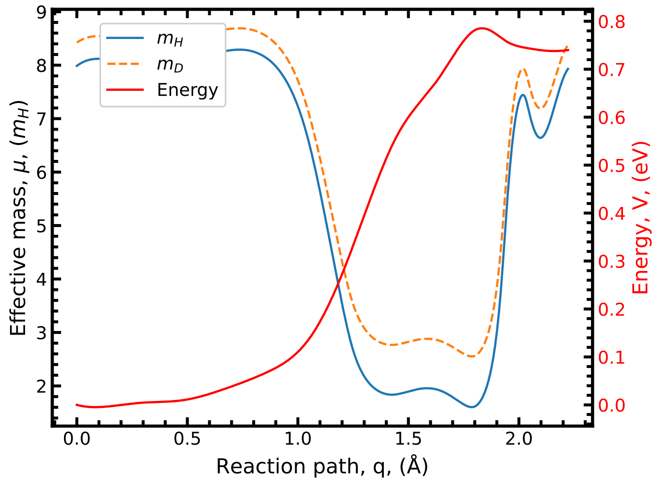

(a) wobble(G-T) $\rightleftharpoons$ G*-T*, $m_p = 1.76 m_H$ and $m_d = 2.69 m_H$

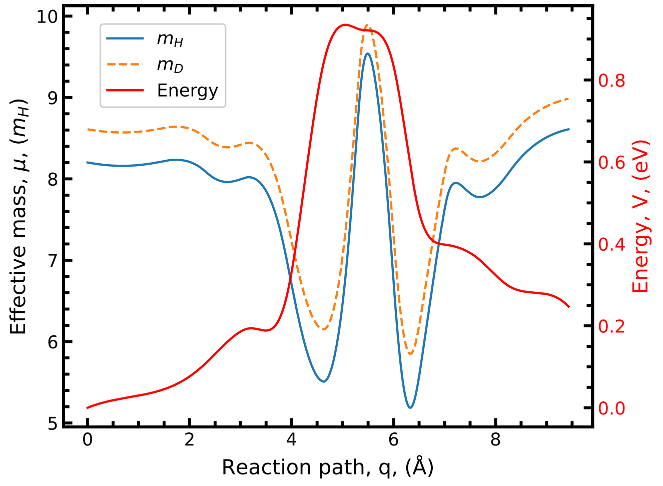

(b) wobble(G-T) $\rightleftharpoons$ G-T*, $m_p = 6.77 m_H$ and $m_d = 7.32 m_H$.

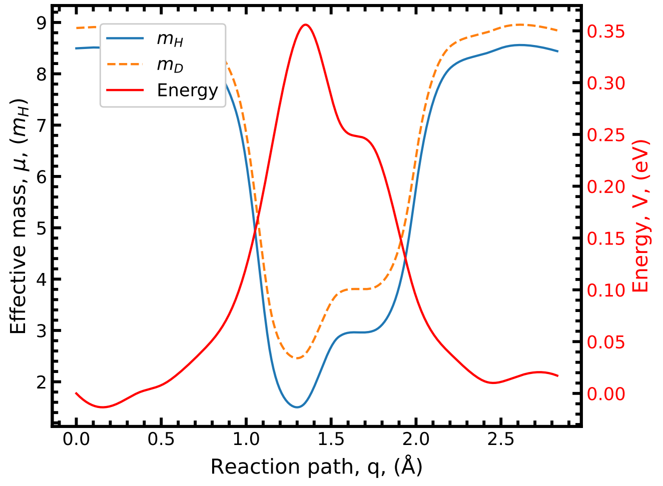

(c) G*-T $\rightleftharpoons$ G-T*, $m_p = 1.59 m_H$ and $m_d = 2.54 m_H$

Supplementary Figure 1: Plots of the effective mass as a function of the reaction path.

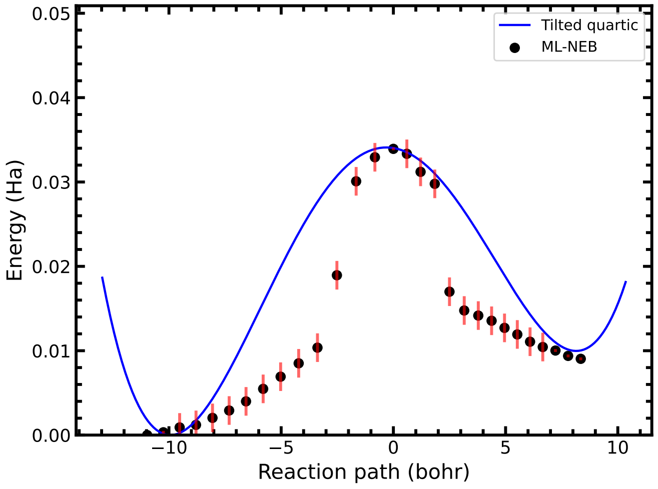

(a) wobble(G-T) ⇌ G-T*

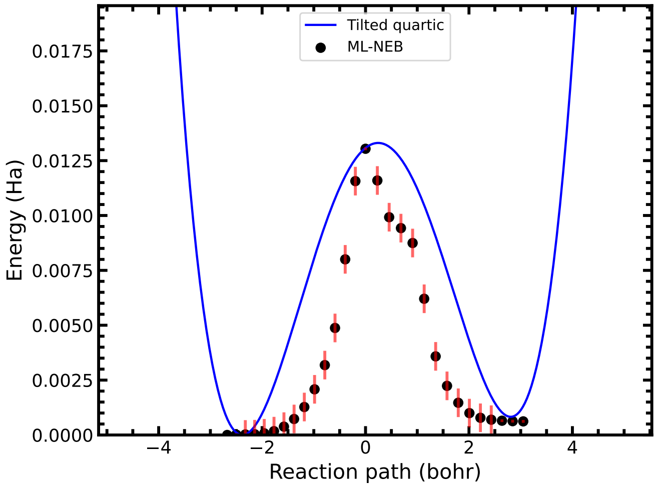

(b) G*-T ⇌ G-T*

Supplementary Figure 2: Fits of the proton transfer potential energy surfaces to the model tilted quartic potential.

into the open quantum systems Hamiltonian and to determine the reactive flux passing through the barrier, we describe the potential energy surface using a tilted quartic double-well model potential given by

$$V(q) = \frac{\hbar\omega_0}{2L_0^2}\, q^2 \left((q - q_0)^2 - \frac{L_0^2}{2} \right) + \frac{\Delta E}{L_0} q \tag{2}$$

Where q is the position coordinate, ω_0 is the effective spring constant of the barrier, L_0 is the displacement between the well minima, q_0 is the additional tilt parameter, and ΔE is the energy difference between the well minima. Each ML-NEB reaction is fitted to this potential to form the following set of potentials shown in Table I. A constrained least-squares fit is performed to the NEB data points; constraining is required to capture the reaction asymmetry and barrier values correctly. This potential is inserted into our open quantum system Hamiltonian; the result is plotted in Fig. 2.

Tunnelling-Ready State: Assuming the Frozen Approximation

Only the inner barrier (section 2 of Fig. 2 in the manuscript) corresponds to the proton transfer between the bases. In contrast, regions 1 and 3 correspond to overall translations of the bases without significant changes in the

Supplementary Table I: Summary of the potential parameters used to describe the proton transfer reactions. The following parameters are defined: ω_0 spring constant of the barrier, L_0 is the displacement, q_0 is the additional tilt parameter, and ΔE well energy.

Parameter	wobble(G-T) $\rightleftharpoons$ G-T*	G*-T $\rightleftharpoons$ G-T*
ω_0	0.001 41 AUT	0.007 60 AUT
L_0	12.76 a_0	3.66 a_0
q_0	−1.40 a_0	0.45 a_0
ΔE	−0.005 56 E_h	0.006 78 E_h

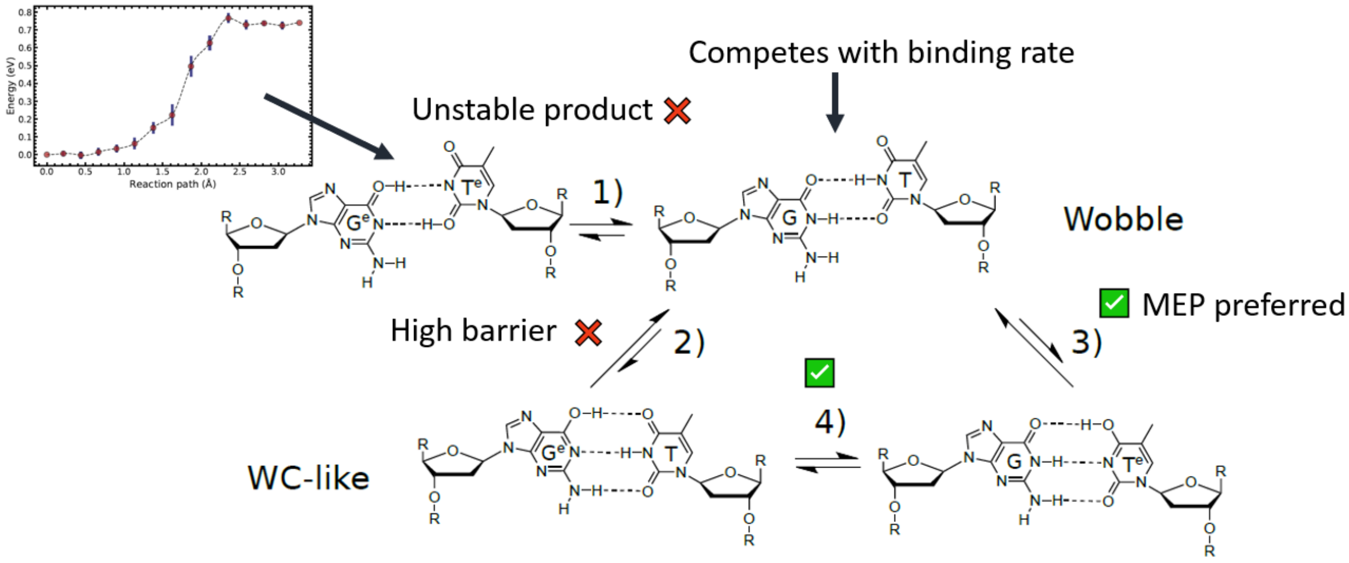

Supplementary Figure 3: Schematic representation of the G-T wobble mispairs and the conversion to a Watson-Crick-like configuration via a proton transfer process.

hydrogen bond length. This observation is compatible with a so-called "tunnelling-ready state" along the reaction path, whereby the hydrogen bonds become partly compressed. To model the state, we take the image corresponding to the start of the proton transfer barrier from the wobble to the Watson-Crick pathway and assume that the local environment has induced this conformation change. Then at this point, the proton transfer timescale would be much quicker due to its low mass than the whole base translating. So at this point, the proton could transfer, and the rest of the base would recoil. The fast proton transfer could facilitate the required rearrangement for the base to snap into a Watson-Crick-like shape. We take the image along the reaction path, constrain all but the hydrogen atom, and calculate the minimum energy path the hydrogen would trace out while transferring across. During the transfer, we neglect rearrangements from the rest of the atoms. The result is a reaction path that may not be the minimum energy pathway but has a higher overall rate due to the boost from tunnelling contributions. The first minimum has an energy difference of 0.621 eV compared to the ground state G-T wobble structure. Assuming this configuration is explored with a penalty in the form $\exp(-E/k_BT)$ gives a weighting of $\sim 3.18 \times 10^{-11}$.

Summary of the Proton Transfer Energy Landscapes

We utilise the methods described before to determine the reaction pathway of the proton transfer in the formation of wobble mismatches. An overview of the reaction pathways is shown in Fig. 3. The results are summarised in table II, and figs. 4 5 6.

Supplementary Table II: Summary of the reactions. With, forward reaction barrier E_f, reverse reaction barrier E_r, reaction asymmetry ΔE, forward reaction free-energy barrier G_f, reverse reaction free-energy barrier G_r, reaction free-energy asymmetry ΔG, and the imaginary frequency at the transition state ν_i.

Parameter	wobble(G-T) $\rightleftharpoons$ wobble(G*-T*)	wobble(G-T) $\rightleftharpoons$ G-T*	G*-T $\rightleftharpoons$ G-T*
E_f	0.766 eV	0.926 eV	0.356 eV
E_r	0.026 eV	0.680 eV	0.339 eV
ΔE	0.740 eV	0.246 eV	0.017 eV
G_f	0.497	0.774 eV	0.075 eV
G_r	-0.191	0.465 eV	0.107 eV
ΔG	0.688	0.309 eV	0.032 eV
ν_i	1022.3 cm^{-1}	156.3 cm^{-1}	1172.9 cm^{-1}

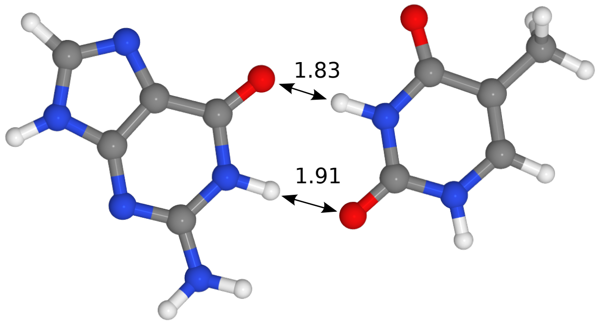

(a) wobble form of G-T

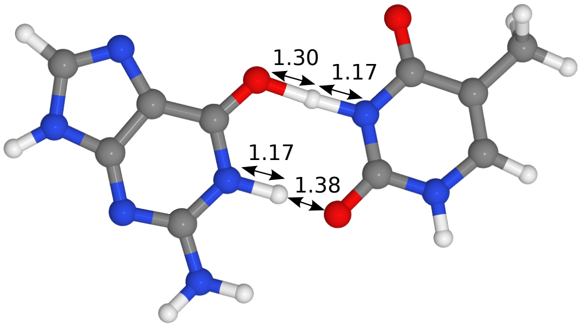

(b) Proton transfer transition state

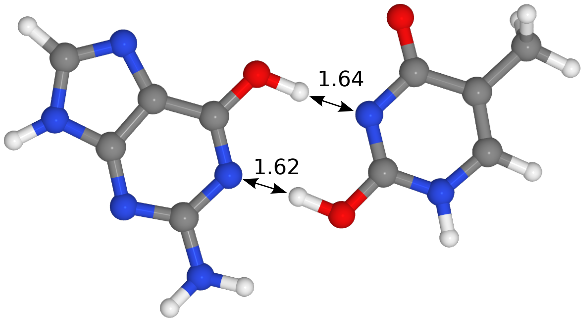

(c) wobble form of G*-T*

Supplementary Figure 4: Optimised geometries of the proton transfer reaction from the canonical wobble forms to the tautomeric wobble configuration, wobble(G-T) $\rightleftharpoons$ wobble(G*-T*). Here the bases do not translate to facilitate proton transfer but instead transfer along the hydrogen bonds, forming a double proton transfer product.

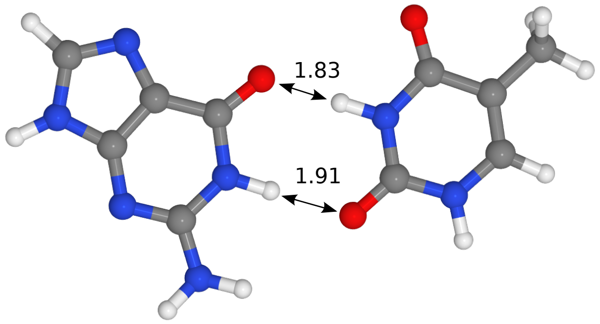

(a) wobble form of G-T

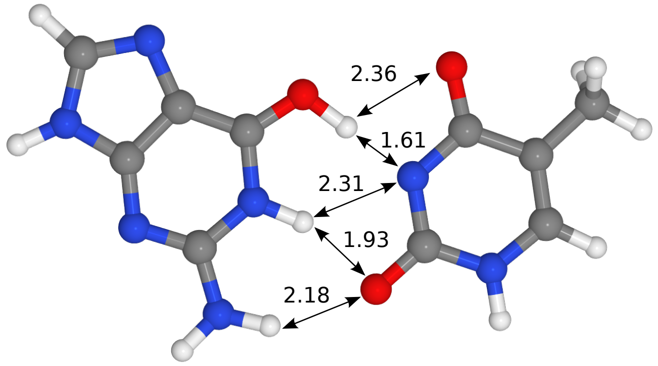

(b) Proton transfer transition state

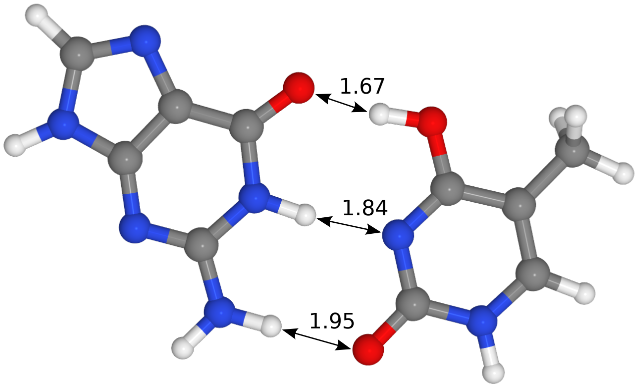

(c) Watson-Crick-like G-T*

Supplementary Figure 5: Optimised geometries of the proton transfer reaction from the canonical wobble forms to the tautomeric Watson-Crick-like configuration, wobble(G-T) $\rightleftharpoons$ G-T*. Here the bases translate relative to each other to facilitate the proton transfer.

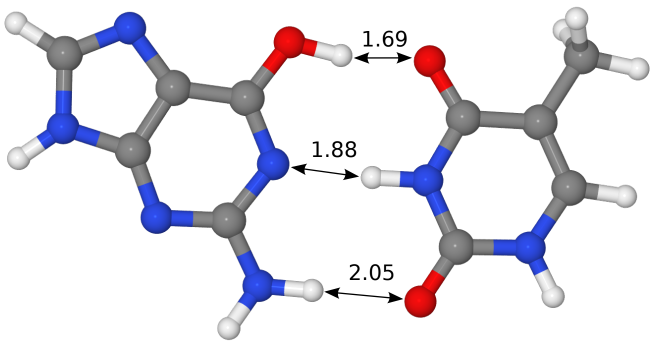

(a) Watson-Crick-like G*-T

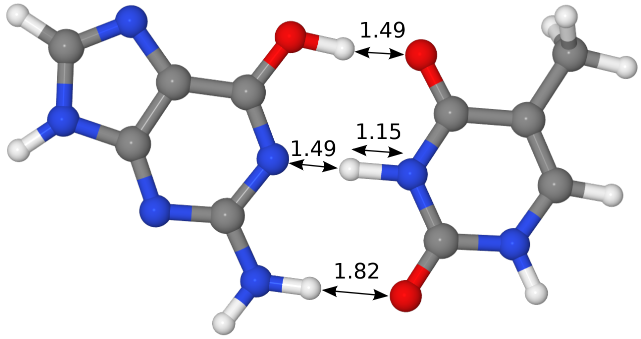

(b) Transition state

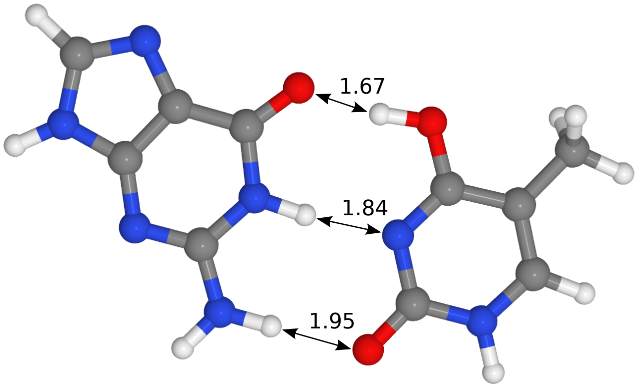

(c) Watson-Crick-like G-T*

Supplementary Figure 6: Optimised geometries of the proton transfer reaction from the Watson-Crick-like forms, G*-T $\rightleftharpoons$ G-T*. Here double proton transfer along the hydrogen bonds swaps the enol form between the two bases.

SUPPLEMENTARY NOTE 2: USING OPEN QUANTUM SYSTEMS TO DESCRIBE TUNNELLING

An ideally isolated quantum system, particularly in biology, is unlikely. Instead, the environment is constantly interacting with the system. In the cellular environment, there is a constant energy flow between the system and the environment through vibrations and collisions with the surrounding solvent and proteins, constantly perturbing the quantum system. Once decoherence sets in, we might expect entirely classical behaviour to emerge. To describe this transition region, we require a theoretical framework to describe the protons in DNA using an open quantum systems approach.

The idea of an open quantum system is to incorporate interactions with the local environment. These interactions significantly change the system's dynamics and result in quantum dissipation and decoherence. The general idea is to couple a system Hamiltonian $\hat{H}_{\mathrm{S}}$ with a bath $\hat{H}_{\mathrm{B}}$ via an interaction $\hat{H}_{\mathrm{I}}$,

$$\hat{H}_{\mathrm{SB}} = \hat{H}_{\mathrm{S}} + \hat{H}_{\mathrm{B}} + \hat{H}_{\mathrm{I}}. \tag{3}$$

Here the interaction term generates quantum and classical correlations between the system and the environment [19].

The Wigner-Moyal Caldeira-Leggett Model

Wigner introduced a quantum-mechanical distribution function in phase space by considering the integral transform of the wave function [20]

$$W(q,p,t) = \frac{1}{\pi\hbar}\int \psi^*(q+q')\psi(q-q')e^{2ipq'/\hbar}dq', \tag{4}$$

or, for a mixed quantum state, using a density matrix,

$$W(q,p,t) = \frac{1}{2\pi\hbar}\int e^{-ipq'/\hbar}\left\langle q+\frac{q'}{2}\right|\tilde{\rho}\left|q-\frac{q'}{2}\right\rangle dq'. \tag{5}$$

The Wigner transformation is the Fourier transform of the antidiagonals of the density matrix when that matrix is expressed in the position basis. Taking the partial time derivative and employing the time-dependent Schrödinger equation, we obtain the Wigner-Moyal (WM) equation describing the time evolution of the Wigner function. Explicitly:

$$\frac{\partial}{\partial t}W(q,p,t) = \mathcal{L}(q,p)\,W(q,p,t). \tag{6}$$

The WM equation is a deterministic dynamical equation that encapsulates the uncertainty in position q and momentum p into a quasi-probability density $W(q,p,t)$ [20, 21]. The quantum Liouvillian ($\mathcal{L}$) for the Wigner function is given by the kinetic ($\mathcal{K}$) and potential ($\mathcal{V}$) terms

$$\mathcal{L}(q,p) \equiv \mathcal{K}(q,p) + \mathcal{V}(q,p). \tag{7}$$

with terms,

$$\mathcal{K}(q,p)W(q,p) \equiv -\frac{p}{m}\frac{\partial}{\partial q}W(q,p) \tag{8}$$

and

$$\mathcal{V}(q,p)W(q,p) \equiv -\frac{i}{\hbar}\left(\mathcal{V}(q)\star W(p,q) - W(p,q)\star\mathcal{V}(q)\right). \tag{9}$$

Here, we use the star operator, $\star$, corresponding to the Moyal product [22, 23],

$$\star \equiv \exp\left[\frac{i}{2}\left(\underset{\leftarrow q}{\partial}\underset{\rightarrow p}{\partial} - \underset{\rightarrow q}{\partial}\underset{\leftarrow p}{\partial}\right)\right]. \tag{10}$$

The directional differentiation operators from the left and right appearing here are defined as

$$\underset{\rightarrow x}{\partial} f(x) = f(x)\underset{\leftarrow x}{\partial} \equiv \frac{\partial f(x)}{\partial x}. \tag{11}$$

Consequently,

$$\mathcal{V}(q,p)W(q,p) \equiv \frac{\partial V}{\partial q}\frac{\partial W}{\partial p} + \sum_{r=1}^{\infty} \frac{(\mathrm{i}\hbar/2)^{2r}}{(2r+1)!}\frac{\partial^{2r+1}V}{\partial q^{2r+1}}\frac{\partial^{2r+1}W}{\partial p^{2r+1}}. \tag{12}$$

For brevity, we drop the dependence and expand the potential terms,

$$\frac{\partial W}{\partial t} = -\frac{p}{m}\frac{\partial W}{\partial q} + \frac{\partial V}{\partial q}\frac{\partial W}{\partial p} - \frac{\hbar^2}{24}\frac{\partial^3 V}{\partial q^3}\frac{\partial^3 W}{\partial p^3} + \mathcal{O}\left(\hbar^4\right). \tag{13}$$

Here the potential terms have been expanded as the Taylor series truncated to just the first two terms, which is frequently done in literature [21]. The series expansion contains powers of $\hbar$ and introduces quantum effects into the dynamics. The equivalent phase-space formulation of Caldeira-Leggett's model, also known as the Wigner-Moyal Caldeira-Leggett (WM-CL) equation, is written as [20, 24]

$$\frac{\partial W}{\partial t} = \underbrace{-\frac{p}{m}\frac{\partial W}{\partial q} + \frac{\partial V}{\partial q}\frac{\partial W}{\partial p} - \frac{\hbar^2}{24}\frac{\partial^3 V}{\partial q^3}\frac{\partial^3 W}{\partial p^3} + \mathcal{O}\left(\hbar^4\right)}_{\text{Schrödinger dynamics}} \underbrace{+\gamma\frac{\partial pW}{\partial p}}_{\text{Dissipation}} \underbrace{+\gamma m k_{\mathrm{B}} T \frac{\partial^2 W}{\partial p^2}}_{\text{Decoherence}}. \tag{14}$$

The WM-CL equation has a similar form to the WM equation [25]; however, it now contains two additional terms describing dissipation and decoherence arising from the coupling to the quantum bath.

Low-temperature Correction

The WM-CL equation is valid in the weak coupling and high-temperature regime $k_{\mathrm{B}}T \gg E_0$, where E_0 is the zero-point energy of the uncoupled system [24]. However, for this system and biologically relevant temperatures ($T \approx 300\,\mathrm{K}$), the high-temperature limit can no longer be a valid approximation. Consequently, we adopt a temperature correction [26–30],

$$T \to \tilde{T} = \frac{\hbar\Omega}{2k_{\mathrm{B}}}\coth\left(\frac{\hbar\Omega}{2k_{\mathrm{B}}T}\right). \tag{15}$$

The correction modifies the temperature of the bath such that it saturates to the zero-point energy of the system at a low temperature, indicating that the lowest energy the system can take is the zero-point energy of the system. Without the correction, the CL model breaks down and fails to maintain the generalised position-momentum uncertainty principle.

$$\lim_{\tilde{T}\to 0^+} \coth\left(\frac{\hbar\Omega}{2k_{\mathrm{B}}T}\right) \to 1. \tag{16}$$

While in the high-temperature limit, it is simply the leading term in the Taylor expansion of $\coth x = \frac{1}{x} + \frac{x}{3} - \frac{x^3}{45} + \cdots$ [24, 26–30],

$$k_{\mathrm{B}}\tilde{T} = \frac{\hbar\Omega}{2}\coth\left(\frac{\hbar\Omega}{2k_{\mathrm{B}}T}\right) \sim k_{\mathrm{B}}T. \tag{17}$$

The addition of the coth term permits using the WM-CL equation at lower temperatures. Within the harmonic approximation, assuming that at the potential global minimum, the third-order or high terms vanish Ω can be approximated by inspecting the second derivative of the potential,

$$\Omega_{\text{approx.}} = \sqrt{m^{-1}\frac{\partial^2 V\left(q_{\text{min.}}\right)}{\partial q^2}} \tag{18}$$

Where $q_{\text{min.}}$ is the location of the global minimum of the potential. However, the correction limits the system to be in potentials with a low anharmonicity [30]. Note that the original coth term in the influence functional contains the ω of the bath, whereas here, we replace it with a property of the potential.

Numerically Solving

To solve the WM-CL (Eq. 14) equation, we use the Smoluchowski limit. In the Smoluchowski (over-damped) limit, when the bath coupling is much greater than the spring constant at the global minimum ($\gamma >> \Omega$), our system becomes over-damped. Assuming that the oscillations in the bath are much faster than the system dynamics, we approach the Smoluchowski limit ($\gamma = 3900\,\mathrm{cm}^{-1} >> \Omega$). In this limit, the bath induces the separation of timescales between the evolution of position and momentum. We can then take

$$P^{\mathrm{QSE}}(q,t) \equiv \int W(p,q,t)\,dp. \tag{19}$$

In the Smoluchowski limit, the Eq. 14 can be rewritten as [31]

$$\frac{\partial}{\partial t} P^{\mathrm{QSE}}(q,t) = \frac{1}{m\gamma}\frac{\partial}{\partial q}\left[\frac{\partial V}{\partial q} + k_{\mathrm{B}}\tilde{T}\frac{\partial}{\partial q}\right] P^{\mathrm{QSE}}(q,t). \tag{20}$$

Eq. 20 can be solved with the method of lines approach. q and p are discretised to a fixed equally spaced lattice with N_q points in range $[q_{\mathrm{min}}, q_{\mathrm{max}}]$ and N_p equally spaced points in range $[p_{\mathrm{min}}, p_{\mathrm{max}}]$. The partial derivatives are expanded using a second-order central finite difference approach. The outer coefficients are set to zero, corresponding to Dirichlet (reflecting) boundary conditions. We use Feagin's 14 explicit Runge-Kutta algorithms to solve for time [32].

Alternatively, to solve the WM-CL (Eq. 14) equation using the method of lines approach. The partial derivatives are expanded using a second-order central finite difference approach. The outer coefficients are set to zero, corresponding to Dirichlet (reflecting) boundary conditions. To integrate the equations in time, we utilise the VCABM5 algorithm [33], an adaptive fifth-order Adams-Moulton method implemented in the DifferentialEquations.jl ecosystem [32]. We find that VCABM5 offers a good trade-off between accuracy and speed.

Obtaining a Quantum Corrected Reaction Rate

The full forward and reverse reaction rate constants, k_{f} and k_{r}, are obtained from,

$$k_{\mathrm{f,r}} = \frac{\kappa}{h\beta} e^{-G_{\mathrm{f,r}}\,\beta} \tag{21}$$

Where $G_{\mathrm{f,r}}$ corresponds to the Gibbs free energy barrier of the forward and reverse reaction barrier, respectively, the tunnelling factor, κ, encapsulates the quantum-to-classical contribution to the rate, incorporating quantum effects such as tunnelling and non-classical reflections thus, if the quantum contribution is negligible $\kappa \to 1$. On the other hand, if quantum effects dominate $\kappa \gg 1$.

We define κ using,

$$\kappa(T) = 1 + \frac{k_f^{\mathrm{QM}}}{k_f^{\mathrm{CL}}} \tag{22}$$

The classical transition state theory value is determined via

$$k_f^{\mathrm{CL}} = \frac{1}{\beta h}\frac{Q^{\neq}}{Q_R} e^{-\beta E_f} \approx \frac{\omega_0}{2\pi} e^{-\beta E_f}. \tag{23}$$

Where $Q^{\neq}$ and Q_R are the reactant and transition state partition functions, and ω_0 is the harmonic constant at the bottom of the reactant well. The second half of the equation follows previous studies which employed a harmonic approximation to the partition functions [34–37]. Finally, k_{CL} does not depend on the bath modes or the friction. Therefore, the classical rates are independent of the friction value.

At thermodynamic equilibrium, in a canonical ensemble, the populations of the reactant and the product regions have the following stationary values

$$P_r^{\mathrm{eq}} = \frac{1}{Q}\int\int W^{\mathrm{eq}}\,(1-\hat{h}(q))\,dp\,dq \tag{24}$$

$$P_p^{\mathrm{eq}} = \frac{1}{Q}\int\int W^{\mathrm{eq}}\,\hat{h}(q)\,dp\,dq. \tag{25}$$

Where $Q = \int\int W^{\rm eq}\, dp\, dq$ partition function for the overall system. $\hat{h}(q)$ is a Heaviside step function that projects onto the product side of a transition state dividing surface (reaction barrier). At thermal equilibrium, $W^{\rm eq}(q,p) = W(q,p,t \to \infty)$, and we can integrate Eq. 14 until it comes to a stationary solution.

In chemical kinetics, this equilibrium can be viewed as dynamically reached in the long-time limit when starting from a non-stationary initial state. If both the forward and backward reactions are governed by rate processes, a relatively simple kinetic equation can be used to describe the population of the reactant (P_r) or the product (P_p)

$$\frac{d}{dt}P_r(t) = -\frac{d}{dt}P_p(t) = -k_f^{\rm QM}(T)P_r(t) + k_r^{\rm QM}(T)P_p(t). \tag{26}$$

Where the thermal rate constants for the forward ($k_f^{\rm QM}$) and reverse ($k_f^{\rm QM}$) reactive processes. Consequently, we have a detailed balance requirement

$$\frac{k_f^{\rm QM}(T)}{k_r^{\rm QM}(T)} = \frac{Q_p(T)}{Q_r(T)}. \tag{27}$$

Where Q_r and Q_p are defined as the reactant and product partition functions

$$Q_r(T) = \int\int W^{\rm eq}\,(1 - \hat{h}(q))\, dp\, dq \tag{28}$$

$$Q_p(T) = \int\int W^{\rm eq}\,\hat{h}(q)\, dp\, dq. \tag{29}$$

We determine the quantum contribution to the chemical reaction rate by monitoring the flux of the density passing through the transition state barrier. We start the system with a non-stationary initial distribution that models the system at thermal equilibrium in the reactant well

$$W(q,p,t=0) = \frac{1}{\mathcal{N}} e^{-\mathcal{H}\tilde{\beta}}(1 - \hat{h}(q)) \tag{30}$$

with $\mathcal{H} = p^2/(2m) + V(q)$ and normalisation constant $\mathcal{N}$. Thus, we monitor the flux of the probability density changes between the left and right-hand well [38–41]

$$\tilde{k}_f^{\rm QM}(t,T) = -\frac{\dot{P}_r(t)}{P_r(t) - [Q_r(T)/Q_p(T)]\,[1 - P_r(t)]} \tag{31}$$

We require that a time-scale separation exists between the reaction and other dynamical processes in the system. After some characteristic time, τ_c [42] the phenomenological rate law can be adopted since the rate plateaus and becomes time-independent

$$k_f^{\rm QM}(T) = \lim_{t \to \tau_c} \tilde{k}_f^{\rm QM}(t,T). \tag{32}$$

The equilibrium constant can be calculated using

$$K_{\rm eq} = \frac{k_f}{k_r} = \exp\left(-\frac{\Delta G}{k_{\rm B}T}\right). \tag{33}$$

Kinetic Isotope Effect

A strong dependence of the reaction rate on the reduced mass of the system could suggest the involvement of tunnelling [43–45]. Consequently, we determine the kinetic isotope effect (KIE) using

$$\text{KIE} = \frac{k_{\rm f,p}^{\rm QM}}{k_{\rm f,d}^{\rm QM}}, \tag{34}$$

where $k_{\rm f,p}^{\rm QM}$ ($k_{\rm f,d}^{\rm QM}$) is the forward rate for a proton (deuteron), obtained from applying Eq. 32.

Supplementary Table III: Summary of the quantum and classical contributions to the reactions. With terms, forward reaction rate k_f, reverse reaction barrier k_r, reactant lifetime τ_f, product lifetime τ_r, chemical equilibrium value K_{eq}, quantum vs classical rate contribution κ, KIE (kinetic isotope effect).

Parameter	wobble(G-T) $\rightleftharpoons$ G-T*	TRS wobble(G-T) $\rightleftharpoons$ G-T*	G*-T $\rightleftharpoons$ G-T*
k_f	$5.244 \times 10^{-1}\,\mathrm{s}^{-1}$	-	$6.090 \times 10^{12}\,\mathrm{s}^{-1}$
k_r	$8.767 \times 10^{4}\,\mathrm{s}^{-1}$	-	$1.753 \times 10^{12}\,\mathrm{s}^{-1}$
τ_f	$1.907\,\mathrm{s}$	-	$1.642 \times 10^{-13}\,\mathrm{s}$
τ_r	$1.141 \times 10^{-5}\,\mathrm{s}$	-	$5.706 \times 10^{-13}\,\mathrm{s}$
K_{eq}	5.982×10^{-6}	-	3.475
κ	1.02	99.00	18.10
KIE	1.10	10.15	4.25

Summary of the Quantum Tunnelling Effects in Proton Transfer

We explore to what degree tunnelling plays a role in each reaction. First, we calculate tunnelling rates on the DFT calculated potentials after fitting them with an analytical function; the supplementary information contains a complete description of the parameters. We then insert this potential into the system Hamiltonian to obtain a tunnelling correction. The results are shown in table III.

Supplementary Table IV: Summary of the potential parameters used to describe the proton transfer reactions. The following parameters are defined: ω_0 spring constant of the barrier, L_0 is the displacement, q_0 is the additional tilt parameter, and ΔE well energy.

Parameter	wobble(G-T) $\rightleftharpoons$ G-T*			G*-T $\rightleftharpoons$ G-T*		
	Aqueous	B-DNA	Poly-λ	Aqueous	B-DNA	Poly-λ
ω_0	0.001 41 AUT	0.001 41 AUT	0.001 41 AUT	0.001 41 AUT	0.001 41 AUT	0.001 41 AUT
L_0	12.76 a_0	12.76 a_0	12.76 a_0	12.76 a_0	12.76 a_0	12.76 a_0
q_0	12.76 a_0	12.76 a_0	12.76 a_0	12.76 a_0	12.76 a_0	12.76 a_0
ΔE	$-0.005\,56\ E_\mathrm{h}$	$-0.005\,56\ E_\mathrm{h}$	$-0.005\,56\ E_\mathrm{h}$	$-0.005\,56\ E_\mathrm{h}$	$-0.005\,56\ E_\mathrm{h}$	$-0.005\,56\ E_\mathrm{h}$

SUPPLEMENTARY NOTE 3: COMPARING THE EFFECT OF THE ENVIRONMENT

Extracting the Free Energy Pathway

To compare how the environment has an impact on the tunnelling, we extract the free energy pathway data from Li *et al.*[46] using WebPlotDigitizer - a web-based tool to extract numerical data from plots [47]. We then have to scale the free energy pathway so that the energy is a function of the reaction path instead of the image index. To do this, we assume that each reaction follows the same path as the ML-NEB data we report. Consequently, we perform a linear rescaling to map the free energy pathway onto our data.

Next, using the extracted free energy pathway, which is now a function of the reaction path, we perform a constrained least-squares fit to adopt the reaction profile into the open quantum systems Hamiltonian. See supplementary note 1 for further details. The result of the fit is summarised in table IV.

Comparing the Free Energy Pathway

Fig. 7 compares our ML-NEB data of the wobble(G-T)$\rightleftharpoons$G-T* (panel a) and G*-T$\rightleftharpoons$G-T* (panel b) reaction with the free energy curve from Li *et al.*[46].

For panel a), the wobble reaction, all reaction paths have a similar initial energy trend, corresponding to the classical sliding and compression of the G-T wobble to facilitate the proton transfer; this mechanism is described in Fig. 2 of the main document. Furthermore, there is little to no variation between the environmental systems suggesting that the initial path is similar irrespective of the local environment. However, our barrier is slightly smaller than the one of the DNA/polymerase system but larger than the isolated B-DNA and DNA/aqueous solution barriers. While our free energy corrected barrier shown in table II is within 14% of the B-DNA. Similarly, the ML-NEB reaction energy matches the B-DNA system within 16%. However, the barrier is significantly higher for the polymerase system, but the reaction energy is much lower. On the other hand, for Fig. 7b), in the Watson-Crick to Watson-Crick reaction, our energy is larger than the free energy profiles. However, with our free energy profiles, our barrier is significantly reduced. Consequently, overall our energy profiles are within reasonable agreement with Li *et al.*[46] B-DNA system.

Comparing Environmental Effects on the Quantum Tunnelling

Finally, we use the extracted free energy potentials to determine the rates due to classical over-the-barrier hopping and tunnelling. The results are summarised in table V. Here, we explore the classical and quantum rates and all the previously calculated parameters.

Due to the wide barrier, we find an insignificant amount of tunnelling for the wobble mechanism for all environments. This finding is consistent with our previous finding regarding our ML-NEB potential. Furthermore, as there is little tunnelling, the KIE is also low, again indicating that the reaction is isotopic independent and predominantly classical. Thus if we adopt the Li *et al.* models and ignore the frozen approximation, as we detailed in supplementary note 1, we conclude that there is little dependence on the choice of environments on the tunnelling and instead, the proton transfer is an over-the-barrier classical behaviour. Here we note that the classical B-DNA rate is consistent with the NMR data [48–50].

On the other hand, the reaction weakly depends on the local environment for the Watson-Crick to Watson-Crick reaction, varying from 2.41 to 4.84. Overall, the quantum-to-classical ratio increases in the polymerase compared to

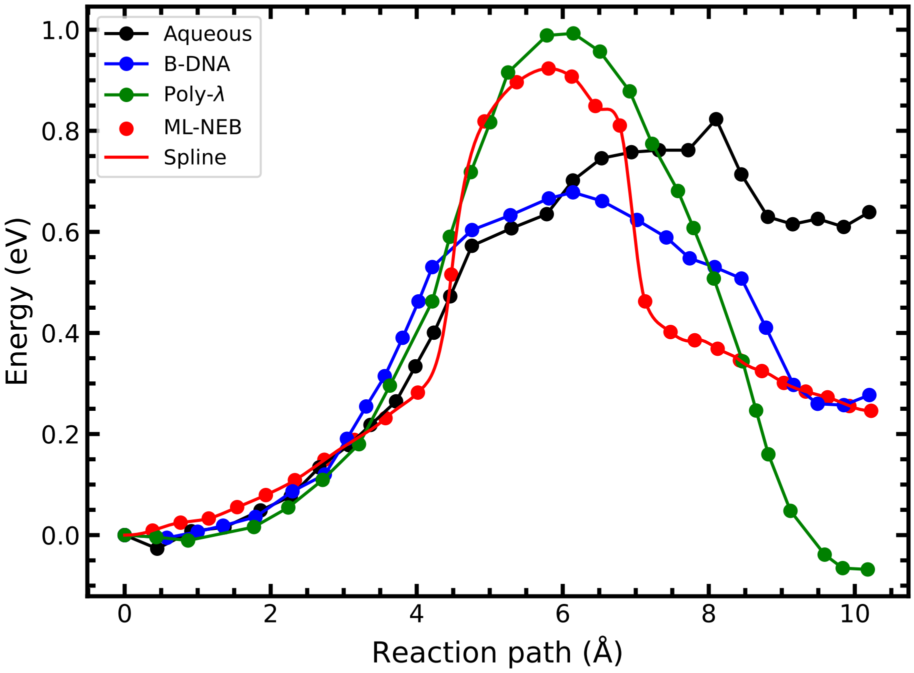

(a) wobble(G-T)⇌G-T*

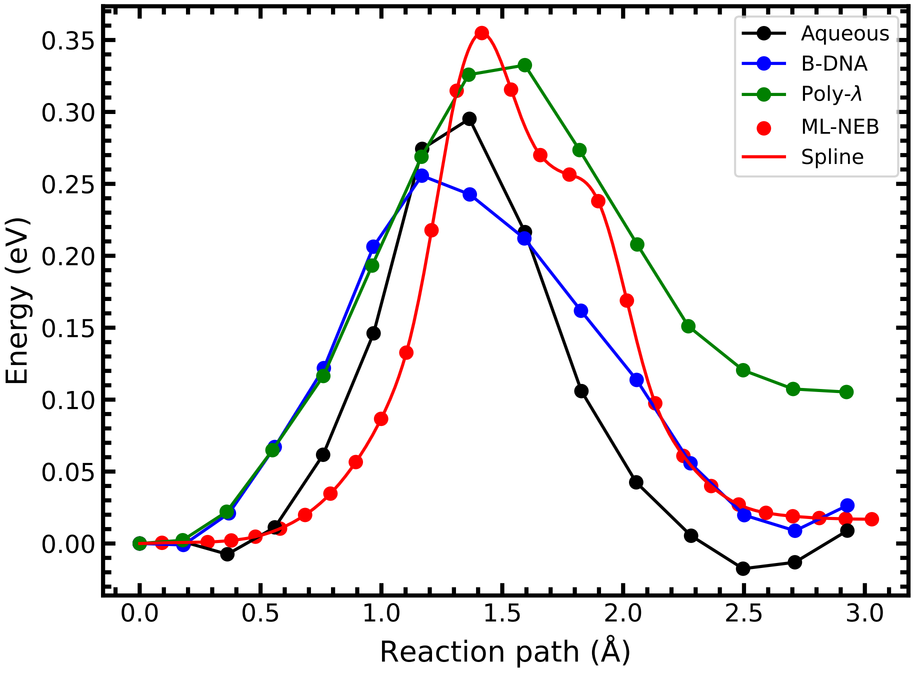

(b) G*-T⇌G-T*

Supplementary Figure 7: Comparison of the Minimum energy paths of wobble(G-T)⇌G-T* and G*-T⇌G-T* reactions. Here, the aqueous, B-DNA, and poly-λ are taken from Li *et al.*[46]. In red is the data obtained using a machine-learning approach to the nudged elastic band method.

Supplementary Table V: Summary of the quantum and classical contributions to the reactions. With terms, forward reaction rate k_f, reverse reaction barrier k_r, reactant lifetime τ_f, product lifetime τ_r, chemical equilibrium value K_{eq}, quantum vs classical rate contribution κ, KIE (kinetic isotope effect).

Parameter	wobble(G-T) $\rightleftharpoons$ G-T*			G*-T $\rightleftharpoons$ G-T*		
	Aqueous	B-DNA	Poly-λ	Aqueous	B-DNA	Poly-λ
k_f	$1.775 \times 10^{-1}\,\text{s}^{-1}$	$8.911\,\text{s}^{-1}$	$3.212 \times 10^{-6}\,\text{s}^{-1}$	$2.493 \times 10^{8}\,\text{s}^{-1}$	$5.959 \times 10^{8}\,\text{s}^{-1}$	$7.494 \times 10^{7}\,\text{s}^{-1}$
k_r	$2.274 \times 10^{10}\,\text{s}^{-1}$	$6.204 \times 10^{5}\,\text{s}^{-1}$	$1.612 \times 10^{-6}\,\text{s}^{-1}$	$2.265 \times 10^{8}\,\text{s}^{-1}$	$7.568 \times 10^{8}\,\text{s}^{-1}$	$4.215 \times 10^{9}\,\text{s}^{-1}$
τ_f	$5.635\,\text{s}^{-1}$	$1.122 \times 10^{-1}\,\text{s}^{-1}$	$3.113 \times 10^{5}\,\text{s}^{-1}$	$4.010 \times 10^{-9}\,\text{s}^{-1}$	$1.678 \times 10^{-9}\,\text{s}^{-1}$	$1.334 \times 10^{-8}\,\text{s}^{-1}$
τ_r	$4.397 \times 10^{-11}\,\text{s}^{-1}$	$1.612 \times 10^{-6}\,\text{s}^{-1}$	$6.205 \times 10^{5}\,\text{s}^{-1}$	$4.415 \times 10^{-9}\,\text{s}^{-1}$	$1.321 \times 10^{-9}\,\text{s}^{-1}$	$2.373 \times 10^{-10}\,\text{s}^{-1}$
K_{eq}	7.804×10^{-12}	1.436×10^{-5}	1.993	1.101	7.874×10^{-1}	1.778×10^{-2}
κ	1.01	1.01	1.03	3.85	2.41	4.84
KIE	1.0	1.0	1.0	2.19	1.67	2.51

the aqueous system due to the classical rate dropping quicker than the quantum rate, as the polymerase system has a higher barrier but a similar width.

SUPPLEMENTARY NOTE 4: QM/MM CALCULATIONS

Ensemble Molecular Dynamics

Classical dynamical simulations were performed in Gromacs 2021.1[51]. A modified input structure was obtained from [46] originating from the experimental crystal structure in PDB entry 3PML[52]. This DNA-enzyme complex contains a wobble(G-T) mismatch involving a 5' thymine as part of a larger DNA molecule and a guanine triphosphate monomer. The topology was generated using the CHARMM36 force field[53, 54], and the SPCE water model [55]. The system was minimised to 12 kJ mol^{-1} nm^{-1} before dynamical NVT simulations were computed with 1 fs timestep at 300 K. The simulation system is shown in 8.

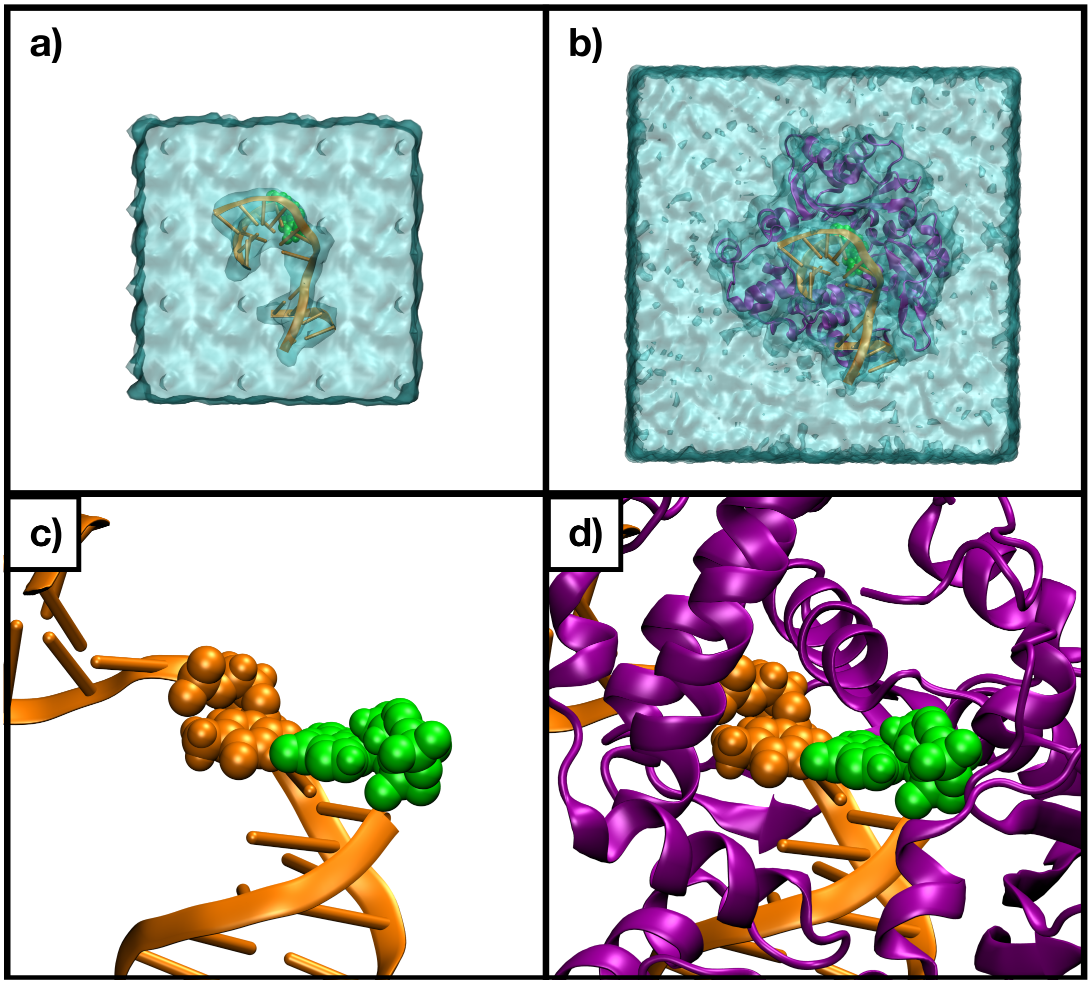

Supplementary Figure 8: The simulation systems for classical MD and hybrid QM/MM MD. Panel a) is the enzyme-less solvated DNA (orange cartoon) system with GTP (green space-filled) bound in the wobble(G-T) configuration, and shown in detail in panel c). Panel b) shows the enzyme-DNA complex of Polymerase-λ (purple cartoon) with the same DNA and GTP representations as panel a). Panel d) shows the wobble(G-T) configuration in the thumb domain of the enzyme.

Ensemble QM/MM MD

Hybrid quantum-classical calculations were performed in Gromacs 2021.1[51], using the interface to quantum chemistry package CP2K[56]. A total of 25 replica QM/MM simulations were performed; per replica, 8000 1 ps timesteps were evaluated within an NVT ensemble at 300 K. For the quantum mechanical region shown in Fig. 9, the BLYP+D3/DZVP-MOLOPT-GTH level of theory was utilised.

Compression reaction coordinate definition

At each timestep of dynamics, whether MM or QM/MM, the distances a, b, and c shown in Figure 9 were recorded. A root-mean-square distance to the tunnelling ready state can be calculated in terms of these three reaction coordinates as shown in Eq. 35. The reference values a_{TRS} are taken from the tunnelling ready state obtained through ML-NEB calculations described previously.

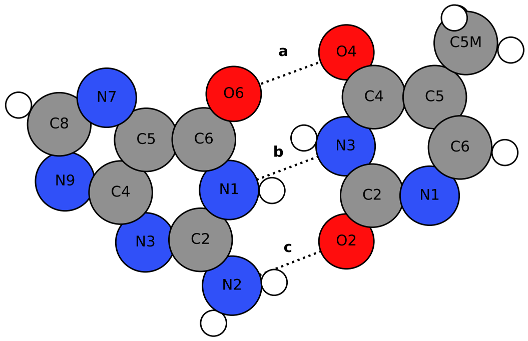

Supplementary Figure 9: Reaction coordinate definition for the statistical sampling of the tunnelling ready state. a,b, and c are the three reaction coordinates used to quantify the compression of the G-T wobble dimer.

$$\Delta = \sqrt{(a - a_{\mathrm{TRS}})^2 + (b - b_{\mathrm{TRS}})^2 + (c - c_{\mathrm{TRS}})^2} \tag{35}$$

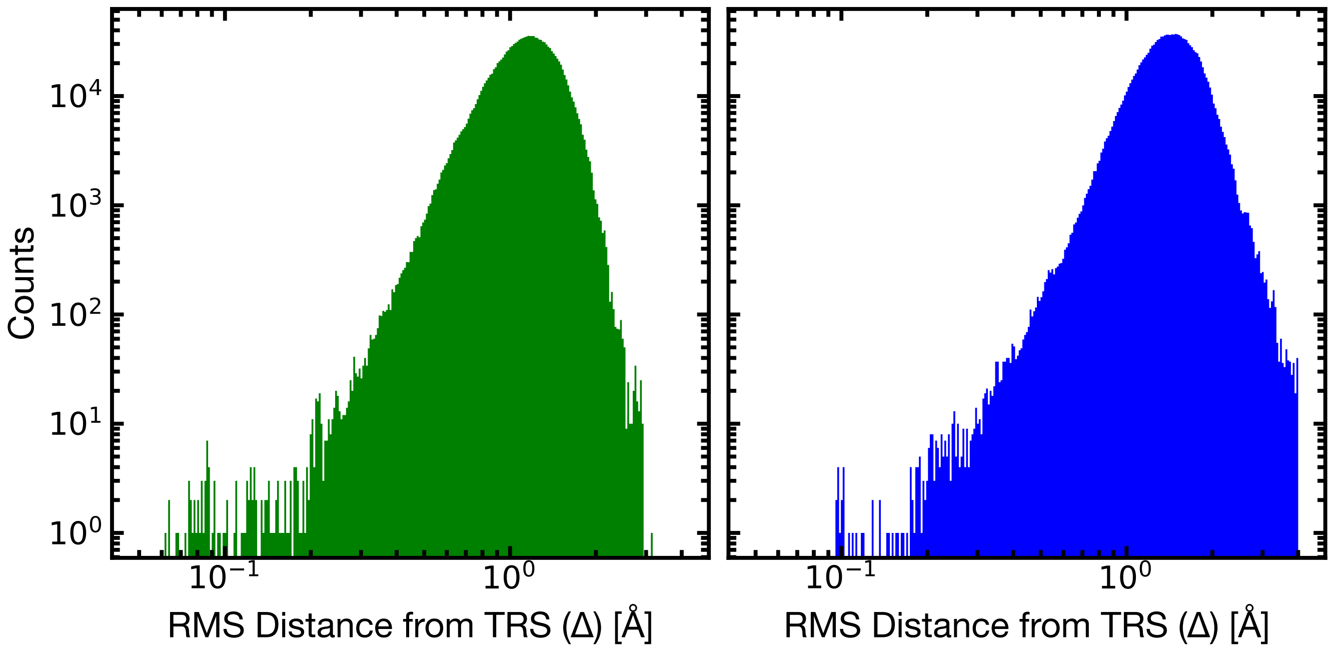

Supplementary Figure 10: Histogram of the compression metric (Δ) for the wobble(G-T) dimer in Polymerase (left panel) and just with DNA (right panel). Each panel corresponds to data aggregated from over 180 ps of ensemble QM/MM MD.

SUPPLEMENTARY REFERENCES

[1] E. Apra, E. J. Bylaska, W. A. De Jong, N. Govind, K. Kowalski, T. P. Straatsma, M. Valiev, H. J. van Dam, Y. Alexeev, J. Anchell, *et al.*, Nwchem: Past, present, and future, The Journal of Chemical Physics **152**, 184102 (2020), https://doi.org/10.1063/5.0004997.

[2] A. D. Becke, Density-functional thermochemistry. iii. the role of exact exchange, The Journal of Chemical Physics **98**, 5648 (1993), https://doi.org/10.1063/1.464913.

[3] S. Grimme, S. Ehrlich, and L. Goerigk, Effect of the damping function in dispersion corrected density functional theory, Journal of Computational Chemistry **32**, 1456 (2011), https://onlinelibrary.wiley.com/doi/pdf/10.1002/jcc.21759.

[4] S. Grimme, J. Antony, S. Ehrlich, and H. Krieg, A consistent and accurate ab initio parametrization of density functional dispersion correction (dft-d) for the 94 elements h-pu, The Journal of Chemical Physics **132**, 154104 (2010), https://doi.org/10.1063/1.3382344.

[5] O. O. Brovarets' and D. M. Hovorun, Atomistic mechanisms of the double proton transfer in the h-bonded nucleobase pairs: Qm/qtaim computational lessons, Journal of Biomolecular Structure and Dynamics **37**, 1880 (2019).

[6] A. Klamt and G. Schüürmann, Cosmo: a new approach to dielectric screening in solvents with explicit expressions for the screening energy and its gradient, J. Chem. Soc., Perkin Trans. 2 , 799 (1993).

[7] D. M. York and M. Karplus, A smooth solvation potential based on the conductor-like screening model, The Journal of Physical Chemistry A **103**, 11060 (1999), https://doi.org/10.1021/jp992097l.

[8] A. V. Marenich, C. J. Cramer, and D. G. Truhlar, Universal solvation model based on solute electron density and on a continuum model of the solvent defined by the bulk dielectric constant and atomic surface tensions, The Journal of Physical Chemistry B **113**, 6378 (2009), pMID: 19366259, https://doi.org/10.1021/jp810292n.

[9] M. H. Hansen, J. A. G. Torres, P. C. Jennings, *et al.*, An atomistic machine learning package for surface science and catalysis, arXiv preprint arXiv:1904.00904 (2019).

[10] J. A. G. Torres, P. C. Jennings, M. H. Hansen, *et al.*, Low-scaling algorithm for nudged elastic band calculations using a surrogate machine learning model, Phys. Rev. Lett. **122**, 156001 (2019).

[11] A. H. Larsen, J. J. Mortensen, J. Blomqvist, *et al.*, The atomic simulation environment—a python library for working with atoms, J. Phys.: Condens. Matter **29**, 273002 (2017).

[12] S. R. Bahn and K. W. Jacobsen, An object-oriented scripting interface to a legacy electronic structure code, Comput. Sci. Eng. **4**, 56 (2002).

[13] P. Jurečka, J. Šponer, J. Černý, and P. Hobza, Benchmark database of accurate (mp2 and ccsd(t) complete basis set limit) interaction energies of small model complexes, dna base pairs, and amino acid pairs, Phys. Chem. Chem. Phys. **8**, 1985 (2006).

[14] D. S. Tikhonov, A simplistic computational procedure for tunneling splittings caused by proton transfer, Structural Chemistry **33**, 351 (2022).

[15] S. Schweiger, B. Hartke, and G. Rauhut, Double proton transfer reactions at the transition from a concerted to a stepwise mechanism: a comparative ab initio study, Physical Chemistry Chemical Physics **7**, 493 (2005).

[16] S. Schweiger and G. Rauhut, Plateau reactions: Double proton-transfer processes with structureless transition states, The Journal of Physical Chemistry A **107**, 9668 (2003).

[17] R. Meyer and H. H. Günthard, General internal motion of molecules, classical and quantum-mechanical hamiltonian, The Journal of Chemical Physics **49**, 1510 (1968).

[18] R. Meyer and H. H. Günthard, Internal rotation and vibration in ch2= ccl–ch2d, The Journal of Chemical Physics **50**, 353 (1969).

[19] H.-P. Breuer and F. Petruccione, *The Theory of Open Quantum Systems* (Oxford University Press on Demand, 2007).

[20] E. Wigner, On the quantum correction for thermodynamic equilibrium, Phys. Rev. **40**, 749 (1932).

[21] J. Weinbub and D. K. Ferry, Recent advances in wigner function approaches, Applied Physics Reviews **5**, 041104 (2018), https://doi.org/10.1063/1.5046663.

[22] J. E. Moyal, Quantum mechanics as a statistical theory, Mathematical Proceedings of the Cambridge Philosophical Society **45**, 99–124 (1949).

[23] K. İmre, E. Özizmir, M. Rosenbaum, and P. F. Zweifel, Wigner method in quantum statistical mechanics, Journal of Mathematical Physics **8**, 1097 (1967), https://doi.org/10.1063/1.1705323.

[24] A. O. Caldeira and A. J. Leggett, Path integral approach to quantum brownian motion, Physica A: Statistical mechanics and its Applications **121**, 587 (1983).

[25] C. Trahan and R. Wyatt, *Quantum Dynamics with Trajectories: Introduction to Quantum Hydrodynamics*, Interdisciplinary Applied Mathematics (Springer New York, 2006).

[26] I. Burghardt and K. B. Møller, Quantum dynamics for dissipative systems: A hydrodynamic perspective, The Journal of chemical physics **117**, 7409 (2002).

[27] G. Agarwal, Brownian motion of a quantum oscillator, Physical Review A **4**, 739 (1971).

[28] F. Haake and R. Reibold, Strong damping and low-temperature anomalies for the harmonic oscillator, Physical Review A **32**, 2462 (1985).

[29] F. Haake, H. Risken, C. Savage, and D. Walls, Master equation for a damped nonlinear oscillator, Physical Review A **34**, 3969 (1986).

[30] K. H. Hughes, Dissipative quantum phase space dynamics on dynamically adapting grids, The Journal of chemical physics **122**, 074106 (2005).

[31] T. Ikeda and Y. Tanimura, Low-temperature quantum fokker–planck and smoluchowski equations and their extension to multistate systems, Journal of Chemical Theory and Computation **15**, 2517 (2019).

[32] C. Rackauckas and Q. Nie, Differentialequations.jl–a performant and feature-rich ecosystem for solving differential equations in julia, J. Open Res. Softw. **5**, 15 (2017).

[33] J. C. Butcher, Numerical methods for ordinary differential equations in the 20th century, J. Comput. Appl. Math **125**, 1 (2000).

[34] M. Topaler and N. Makri, Quantum rates for a double well coupled to a dissipative bath: Accurate path integral results and comparison with approximate theories, The Journal of Chemical Physics **101**, 7500 (1994), https://doi.org/10.1063/1.468244.

[35] A. Pomyalov and D. J. Tannor, The non-markovian quantum master equation in the collective-mode representation: Application to barrier crossing in the intermediate friction regime, The Journal of Chemical Physics **123**, 204111 (2005), https://doi.org/10.1063/1.2121649.

[36] I. R. Craig, M. Thoss, and H. Wang, Proton transfer reactions in model condensed-phase environments: Accurate quantum dynamics using the multilayer multiconfiguration time-dependent hartree approach, The Journal of Chemical Physics **127**, 144503 (2007), https://doi.org/10.1063/1.2772265.

[37] S. Y. Kim and S. Hammes-Schiffer, Hybrid quantum/classical molecular dynamics for a proton transfer reaction coupled to a dissipative bath, The Journal of chemical physics **124**, 244102 (2006).

[38] Y. Tanimura and P. G. Wolynes, Quantum and classical fokker-planck equations for a gaussian-markovian noise bath, Phys. Rev. A **43**, 4131 (1991).

[39] Y. Tanimura and P. G. Wolynes, The interplay of tunneling, resonance, and dissipation in quantum barrier crossing: A numerical study, The Journal of chemical physics **96**, 8485 (1992).

[40] J. Zhang, R. Borrelli, and Y. Tanimura, Proton tunneling in a two-dimensional potential energy surface with a non-linear system–bath interaction: Thermal suppression of reaction rate, The Journal of Chemical Physics **152**, 214114 (2020).

[41] A. Ishizaki and Y. Tanimura, Multidimensional vibrational spectroscopy for tunneling processes in a dissipative environment, The Journal of chemical physics **123**, 014503 (2005).

[42] P. Hänggi, P. Talkner, and M. Borkovec, Reaction-rate theory: fifty years after kramers, Reviews of modern physics **62**, 251 (1990).

[43] J. P. Klinman and A. R. Offenbacher, Understanding biological hydrogen transfer through the lens of temperature dependent kinetic isotope effects, Accounts of chemical research **51**, 1966 (2018).

[44] L. O. Johannissen, S. Hay, and N. S. Scrutton, Nuclear quantum tunnelling in enzymatic reactions – an enzymologist's perspective, Phys. Chem. Chem. Phys. **17**, 30775 (2015).

[45] L. O. Johannissen, A. I. Iorgu, N. S. Scrutton, and S. Hay, What are the signatures of tunnelling in enzyme-catalysed reactions?, Faraday Discuss. **221**, 367 (2020).

[46] P. Li, A. Rangadurai, H. M. Al-Hashimi, and S. Hammes-Schiffer, Environmental effects on guanine-thymine mispair tautomerization explored with quantum mechanical/molecular mechanical free energy simulations, Journal of the American Chemical Society **142**, 11183 (2020), pMID: 32459476, https://doi.org/10.1021/jacs.0c03774.

[47] A. Rohatgi, Webplotdigitizer: Version 4.6 (2022).

[48] I. J. Kimsey, E. S. Szymanski, W. J. Zahurancik, A. Shakya, Y. Xue, C.-C. Chu, B. Sathyamoorthy, Z. Suo, and H. M. Al-Hashimi, Dynamic basis for dg• dt misincorporation via tautomerization and ionization, Nature **554**, 195 (2018).

[49] I. J. Kimsey, K. Petzold, B. Sathyamoorthy, Z. W. Stein, and H. M. Al-Hashimi, Visualizing transient watson–crick-like mispairs in dna and rna duplexes, Nature **519**, 315 (2015).

[50] A. Rangadurai, E. S. Szymanski, I. Kimsey, H. Shi, and H. M. Al-Hashimi, Probing conformational transitions towards mutagenic watson–crick-like g· t mismatches using off-resonance sugar carbon r 1ρ relaxation dispersion, Journal of Biomolecular NMR , 1 (2020).

[51] H. Bekker, H. Berendsen, E. Dijkstra, S. Achterop, R. Vondrumen, D. Vanderspoel, A. Sijbers, H. Keegstra, and M. Renardus, Gromacs-a parallel computer for molecular-dynamics simulations, in *4th International Conference on Computational Physics (PC 92)* (World Scientific Publishing, 1993) pp. 252–256.

[52] K. Bebenek, L. C. Pedersen, and T. A. Kunkel, Replication infidelity via a mismatch with watson–crick geometry, Proceedings of the National Academy of Sciences **108**, 1862 (2011).

[53] K. Hart, N. Foloppe, C. M. Baker, E. J. Denning, L. Nilsson, and A. D. MacKerell Jr, Optimization of the charmm additive force field for dna: Improved treatment of the bi/bii conformational equilibrium, Journal of chemical theory and computation **8**, 348 (2012).

[54] R. B. Best, X. Zhu, J. Shim, P. E. Lopes, J. Mittal, M. Feig, and A. D. MacKerell Jr, Optimization of the additive charmm all-atom protein force field targeting improved sampling of the backbone ϕ, ψ and side-chain $\chi 1$ and $\chi 2$ dihedral angles, Journal of chemical theory and computation **8**, 3257 (2012).

[55] H. Berendsen, J. Grigera, and T. Straatsma, The missing term in effective pair potentials, Journal of Physical Chemistry **91**, 6269 (1987).

[56] T. D. Kühne, M. Iannuzzi, M. Del Ben, V. V. Rybkin, P. Seewald, F. Stein, T. Laino, R. Z. Khaliullin, O. Schütt, F. Schiffmann, *et al.*, Cp2k: An electronic structure and molecular dynamics software package-quickstep: Efficient and accurate electronic structure calculations, The Journal of Chemical Physics **152**, 194103 (2020).
