# Supplementary material for: Quantum Tunnelling Effects in the Guanine-Thymine Wobble Misincorporation via Tautomerism
Source: J Phys Chem Lett. 2022 Dec 23;14(1):9–15. doi: 10.1021/acs.jpclett.2c03171 (PMC9841559; doi:10.1021/acs.jpclett.2c03171)
Supplement: Supplementary file 2 — jz2c03171_si_002.pdf [file jz2c03171_si_002.pdf]

jz-2022-03171h.R1

Name: Peer Review Information for "Quantum Tunnelling Effects in the Guanine-Thymine Wobble Misincorporation via Tautomerisation"

First Round of Reviewer Comments

Reviewer: 1

Comments to the Author

Proton transfer reactions in DNA and the impact of the associated rare tautomeric forms have been extensively assessed since Löwding proposed that hypothesis in the early 1960s. Theory has been also applied to further understand the role played by such rare tautomeric forms in the observed spontaneous mutation in DNA. First models were based on simple formic acid dimers. More refined (realistic) chemical models (i.e., larger genetic fragments, surrounding media effects) are now available in the literature

Slocombe and co-workers have recently contributed to this field by using the same theoretical approach to related Watson-Crick base pairs (#Ref. 14 Quantum and classical effects in DNA point mutations: Watson–Crick tautomerism in AT and GC base pairs; #Ref. 15: An open quantum systems approach to proton tunneling). Aiming at providing a fair recommendation, this referee has reviewed both the present manuscript as well as these two earlier works (and other works in the bibliography).

There is consequently nothing wrong in the proposed methodology, which has been published in that references. However, it would have been more desirable to have all such accumulated evidence in one publication only, a requisite to deliver a more general view of mutation upon proton transfer phenomena. Over-slicing (i.e., one publication per base pair) reduces novelty.

In the current form, the scope of the manuscript is too narrow to extract real biological conclusions, as it should be seen as an incremental work from the work by Brovarets and co-workers.

The selected chemical system is also a major drawback. As correctly stated by the authors, the polymerase active site is a critical source of replication errors that might lead to genetic mutations. In other words, these mispairs appear at the polymerase active site, but authors used an isolated base pair. The impact of the pockets is completely missing, so that numeric values cannot be extrapolated nor compared to real conditions.

In short, I cannot recommend this paper for publication in the Journal of Physical Chemistry Letters because three major drawbacks:

1. The manuscript does not match the requirement of urgency. It is an incremental work from previous publications, e.g., the use of the same methodology for an additional base pair.

2. This is a too narrow view for a complex phenomenon. Authors must compare these values with other proton transfer equilibria in competition during mutation.

3. The chemical system allows for unrealistic conformation. These mutations appear in the polymerase binding site. The model based on an isolated bases pairs must be improved. Biological media might also impact.

My recommendation is to expand the manuscript to a Regular Paper. Without additional calculations, but as regular contribution, the manuscript might be suitable for ACS Omega. With additional work (more refined model systems and a systematic comparison with all other sources of mutations due to rare tautomers) the paper might be suitable for the Journal of Physical Chemistry A or B.

With regret, I recommend rejection for the submission to JPC Lett.

Reviewer: 2

Comments to the Author

This manuscript describes a way to determine the reaction pathway of several reactions for generating tautomers of the G-T wobble mispair. The authors account for the bases' decoherent and dissipative local environment and identified quantum and classical contributions to the reaction rates by an open quantum systems approach.

And the authors found that the reaction for the wobble(G-T) ↔ G*-T proceeds via a stepwise process . I thought it was a very interesting result.

1) p6

The reader would like to know how much the fifth and sixth terms on the right side of Equation 1, which represent the interaction between DNA and its environment, affected the reaction in the results of this calculation. This would show that the environment surrounding the DNA plays an important role in the wobble(G-T)↔G-T* reaction.

Please explain the ratio of contributions of the Schrödinger dynamics term, the dissipation term, and the decoherence term, respectively.

2) SUPPLEMENTARY NOTE 2

The authors state in SUPPLEMENTARY NOTE 2 that dissipation and decoherence are derived from the coupling to the quantum bath. Where is this quantum bath derived from in DNA? I think this explanation will convince biophysicists.

Author's Response to Peer Review Comments:

Quantum Tunnelling Effects in the Guanine-Thymine Wobble Misincorporation via Tautomerisation

L. Slocombe, M. Winokan, J. S. Al-Khalili, M. Sacchi

December 16, 2022

Thank you for giving us the opportunity to submit a revised draft of the manuscript "Quantum Tunnelling Effects in the Guanine-Thymine Wobble Misincorporation via Tautomerisation". We sincerely thank both of the referees for their comprehensive reports. The level of detail in the referee's responses is extremely appreciated, as is their time and effort to provide insightful comments and suggestions. In light of the concerns raised by the referees, we have significantly improved the content and structure of the paper. Aided and motivated by the referees' comments, we have improved the impact and the quality of the presentation of the data. We have incorporated all their suggestions and addressed each comment point by point.

We have typeset parts of the referees' comments to clarify our responses. In addition, we have highlighted the changes we have made to address their comments. Outlined below are our responses and changes to the paper in line with their suggestions. Our response is formatted in the following way:

The referees' comments are in italics and coloured blue.

Response Included below, our response is written unindented adjacent to the bold lettering.

- Explanation and location of the changes are shown here.
  The changes made to the revised manuscript are in red.

1 Referee 1

Recommendation: While the work is good and publishable, a more appropriate journal is recommended such as ACS Omega, JPCA

Proton transfer reactions in DNA and the impact of the associated rare tautomeric forms have been extensively assessed since Löwding proposed that hypothesis in the early 1960s. Theory has been also applied to further understand the role played by such rare tautomeric forms in the observed spontaneous mutation in DNA. First models were based on simple formic acid dimers. More refined (realistic) chemical models (i.e., larger genetic fragments, surrounding media effects) are now available in the literature

Slocombe and co-workers have recently contributed to this field by using the same theoretical approach to related Watson-Crick base pairs (Ref. 14 Quantum and classical effects in DNA point mutations: Watson–Crick tautomerism in AT and GC base pairs; Ref. 15: An open quantum systems approach to proton tunneling). Aiming at providing a fair recommendation, this referee has reviewed both the present manuscript as well as these two earlier works (and other works in the bibliography).

There is consequently nothing wrong in the proposed methodology, which has been published in that references. However, it would have been more desirable to have all such accumulated evidence in one publication only, a requisite to deliver a more general view of mutation upon proton transfer phenomena. Over-slicing (i.e., one publication per base pair) reduces novelty.

In the current form, the scope of the manuscript is too narrow to extract real biological conclusions, as it should be seen as an incremental work from the work by Brovarets and co-workers.

The selected chemical system is also a major drawback. As correctly stated by the authors, the polymerase active site is a critical source of replication errors that might lead to genetic mutations. In other words, these mispairs appear at the polymerase active site, but authors used an isolated base pair. The impact of the pockets is completely missing, so that numeric values cannot be extrapolated nor compared to real conditions.

In short, I cannot recommend this paper for publication in the Journal of Physical Chemistry Letters because three major drawbacks:

1. The manuscript does not match the requirement of urgency. It is an incremental work from previous publications, e.g., the use of the same methodology for an additional base pair.

2. This is a too narrow view for a complex phenomenon. Authors must compare these values with other proton transfer equilibria in competition during mutation.

3. The chemical system allows for unrealistic conformation. These mutations appear in the polymerase binding site. The model based on an isolated bases pairs must be improved. Biological media might also impact.

My recommendation is to expand the manuscript to a Regular Paper. Without additional calculations, but as regular contribution, the manuscript might be suitable for ACS Omega. With additional work (more refined model systems and a systematic comparison with all other sources of mutations due to rare tautomers) the paper might be suitable for the Journal of Physical Chemistry A or B. With regret, I recommend rejection for the submission to JPC Lett.

Response We thank Referee 1 for their interest in the manuscript and detailed review. We commend the reviewer for further reviewing Ref. 14 and Ref. 15 to provide a critical but fair review. We will now highlight the following key improvements made to the revised manuscript as motivated by the referee's comments:

- We have performed QM/MM calculations on how often the proposed tunnelling ready state is sampled in the polymerase vs the DNA environment.
- Provided further discussion on how the larger environment plays a role.
- Directly compared the effect of the environment on the tunnelling rates.

1.1 Point 1 - Manuscript is too narrow to extract biological conclusions

Here the reviewer raises the issue of novelty and suggests that the methodology has been covered before in previous literature:

> *There is consequently nothing wrong in the proposed methodology, which has been published in that references. However, it would have been more desirable to have all such accumulated evidence in one publication only, a requisite to deliver a more general view of mutation upon proton transfer phenomena. Over-slicing (i.e., one publication per base pair) reduces novelty.*
>
> *In the current form, the scope of the manuscript is too narrow to extract real biological conclusions, as it should be seen as an incremental work from the work by Brovarets and co-workers.*

This follows with the first major drawback raised by the reviewer:

> *1. The manuscript does not match the requirement of urgency. It is an incremental work from previous publications, e.g., the use of the same methodology for an additional base pair.*

Response We thank the reviewer for their robust and open response. However, we believe that we can convince them that this paper reports novel and urgent results and that we are justified in pursuing the publication of our work in the Journal of Physical Chemistry Letters.

- Firstly, the methodology in the paper has evolved from our early works [1,2]. In this letter, we adopted more sophisticated and improved techniques for calculating quantum corrected rates. See supplementary notes 1 and 2. Motivated by the reviewers' comments, we reworked the supplementary information document.
  To clarify the nature of the conducted DFT calculations, we overhauled the content of supplementary note 1. Likewise, to improve clarity, we reworked the contents of supplementary note 2. In addition, we merged the other supplementary notes into the first two and streamlined the content to highlight the methods' novelty. Furthermore, the comments and changes requested by reviewer 2 (localised to supplementary note 2) will be of interest. Here, we provided further explanation of the origin of the bath and how the quantum system interacts with the environment.
- Secondly, regarding the suggested lack of novelty in our results, we must stress that: 1) The role of quantum tunnelling has yet to be explored; thus, this system has never previously reported quantum-corrected rates. 2) The mechanism presented here is vastly different from our other papers, where we considered Watson-Crick base pairs solely and not the wobble mismatch. 3) In this letter, we propose a novel mechanism - tunnelling ready state - which has never been proposed for this system. 4) NMR experiments [3–5] report KIE values and kinetic rates in urgent need of comparison with computational models of the system. 5) To address the possibility of point mutations, it is important to understand DNA base pairs' physiochemistry properties. Furthermore, the G-T wobble mechanism has been previously recognised as a significantly important topic, with papers addressing the issue in high-impact journals such as PNAS [6] and Nature [3, 4, 7]. Most of these studies have been experimental and many questions remain on the theoretical side. In light of this, we updated the paper's first paragraph and added further references.
  ... While there are other sources of replication errors, the fidelity of replication primarily depends on the ability of polymerases to select and incorporate the correct complementary base (see Fig. 1) and reject these wobble mispairs. However, it is proposed [3, 4, 6–11] that the wobble mismatch can form alternative tautomeric configurations that can mimic the WC geometry and lead to erroneous DNA base matches ...
- Thirdly, the reviewer recommends collecting all proton transfer mechanisms into one paper. We believe that it was not in the best interests of the field to delay the publication of what we see as important results. We are also convinced that, in the interests of clarity, we did not wish to cover a full discussion of Watson-Crick tautomerism, classic-versus-quantum proton transfer, density matrix formalism for open quantum systems and DNA separation dynamics all in a single paper, which would have rendered it far too long – and probably confusing. Furthermore, covering all the DNA point-mutation mechanisms (for the first time) in one paper reduces both the readability and the impact of the publication. Not publishing for several years and collecting into one large paper could result in a very large paper which could lead to obscuring the core narrative of each distinct proton transfer mechanism. However, we see the reviewer's point about over-slicing the story. We agree that a balance must be found, but in this circumstance, we are well justified in publishing this work.
- Stimulated by the point raised, we probed how we could provide further evidence of the tunnelling-ready state, which is one of the core mechanisms we postulate. If we can do some statistical analysis on the compression of the G-T wobble mismatch in the polymerase active site that we describe and assume as a classical rearrangement of the bases along the reaction path found before and the proton transfer. We then give credence to the tunnelling ready state, which assumes that there is a separation of timescales between the classical sliding and rearrangement and the proton transfer event. In light of the reviewer's comment,

we performed QM/MM MD calculations, where the inner pocket is treated with QM, and the rest of the system (polymerase and solvent) is treated with MM. The changes can be found on pages 9-11 of the main document.

We now focus on explicitly how the polymerase active site interacts with the G-T wobble mismatch and the tunnelling-ready state. Free energy pathway calculations from Li *et al.* [8] suggest that the polymerase introduces a 46% increase in the proton transfer reaction barrier. However, as the DFT calculations show, for the wobble(G-T)$\rightleftharpoons$G-T* reaction to occur, the nucleotide dimer must first be compressed into a "tunnelling-ready" state. Therefore, it is desirable to know whether this state is populated in a biologically relevant thermal ensemble. To this end, hybrid quantum-classical Quantum-Mechanical/Molecular-Mechanical Molecular-Dynamics (QM/MM MD) simulations were performed, wherein the entire polymerase enzyme and solvent are included explicitly in the simulation system. Many short replica simulations were computed from the wobble configuration obtained through a crystal structure, totalling over 2800 ps of QM/MM MD. This investigation was repeated without the enzyme to highlight the compressing effect of the enzyme's "thumb" region. Both simulation systems are described and illustrated in supplementary note 4.

A metric for the overlap distance between the simulation snapshot and the "tunnelling-ready" state from the ML-NEB calculations is defined as Δ. The metric allows the frequency of how often the state is populated in a biologically relevant thermal ensemble to be determined. Using these QM/MM MD simulations, a cumulative histogram of this data is shown in Fig. 1 for both aqueous DNA and the polymerase DNA complex. Full computational details are provided in supplementary note 4.

The MD simulations demonstrate that the G-T dimer remains in either a wobble configuration consistent with the reactant configuration or exists in a transitory unbound state (see the schematic representation in Fig. 1). Among all the QM/MM MD replica simulations, 0.003% of the trajectory was within 0.096 Å root-mean-squared distance from the "tunnelling-ready" state for the enzyme-DNA complex. Without the enzyme, no Δ values below 0.096 were observed. The lower delta regime corresponding to the TRS was informed by considering the Δ difference between the 5th and 6th ML-NEB data points from Fig. 2. Assuming a uniform distribution of events, this is equivalent to the dimer compressing once every 35.2 ps in the polymerase active site. Despite the approximately 0.275 eV energetic penalty to compression shown in Fig. 2, our dynamical simulations show that this state is reachable within a realistic biological environment. Crucially, without the presence of the enzyme, no population was found below $\Delta = 0.97$ Å, suggesting that the enzyme facilitates the compression. These results justify performing proton transfer calculations for the wobble(G-T)$\rightleftharpoons$G-T* reaction from such a compressed "tunnelling-ready" state as shown in Fig. 3. While the proton transfer mechanism starts from a more compressed G-T wobble conformation, reaction rate calculations must now also consider the sparsity with which this compressed state is observed, as the barrier shown in Fig. 3 is only accessible less than 0.003% of the time.

- Furthermore, we updated the supplementary information detailing the QM/MM methods. See the new section; supplementary note 4.

Ensemble Molecular Dynamics

Classical dynamical simulations were performed in Gromacs 2021.1 [12]. A modified input structure was obtained from [8] originating from the experimental crystal structure in PDB entry 3PML [13]. This DNA-enzyme complex contains a wobble(G-T) mismatch involving a 5' thymine as part of a larger DNA molecule and a guanine triphosphate monomer. The topology was generated using the CHARMM36 force field [14,15], and the SPCE water model [16]. The system was minimised to 12 $\mathrm{kJ\,mol^{-1}\,nm^{-1}}$ before dynamical NVT simulations were computed with 1 fs timestep at 300 K. The simulation system is shown in 2.

Ensemble QM/MM MD

Hybrid quantum-classical calculations were performed in Gromacs 2021.1 [12], using the interface to quantum chemistry package CP2K [17]. A total of 25 replica QM/MM simulations were performed; per replica, 8000 1 ps timesteps were evaluated within an NVT ensemble at 300 K. For the quantum mechanical region shown in Fig. 3, the BLYP+D3/DZVP-MOLOPT-GTH level of theory was utilised.

Compression reaction coordinate definition

At each timestep of dynamics, whether MM or QM/MM, the distances a, b, and c shown in Figure 3 were recorded. A root-mean-square distance to the tunnelling ready state can be calculated in terms of these

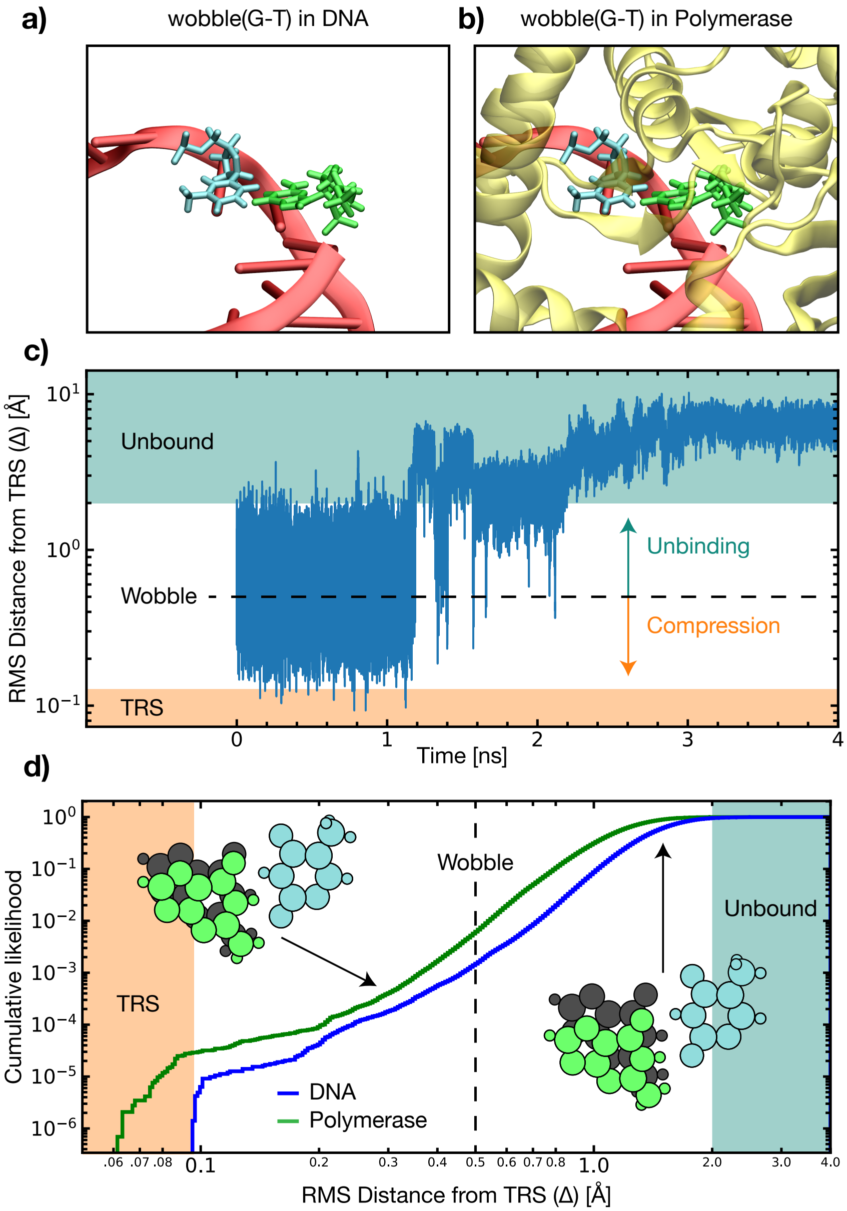

Figure 1: Dynamical investigation into the biological relevance of the compressed "tunnelling-ready" state (TRS) of the wobble(G-T) mismatch. The compression of the wobble(G-T) mismatch is considered in a DNA insertion site with the polymerase enzyme (panel b)) and without the enzyme (panel a)). An RMS distance is defined to the TRS and plotted c) during a single long molecular dynamics trajectory and d) aggregated from over 2800 ps of QM/MM MD simulations. In panel c), the RMS distance to the wobble(G-T) configuration is shown as a black dashed line, and two additional regimes are illustrated. Firstly, an unbound regime is defined with $\Delta > 2.0$ Å, and a set of "tunnelling-ready"/compressed states with $\Delta < 0.096$ Å. In panel d), the cumulative likelihood across a range of Δ values is graphed for the Polymerase-DNA complex (green line) and aqueous DNA (blue line). In this context, the cumulative likelihood determines the probability of finding the dimer at an RMS distance below the given value. Two example conformations are shown relative to the TRS (grey circles) position.

three reaction coordinates as shown in Eq. 1. The reference values a_{TRS} are taken from the tunnelling ready state obtained through ML-NEB calculations described previously.

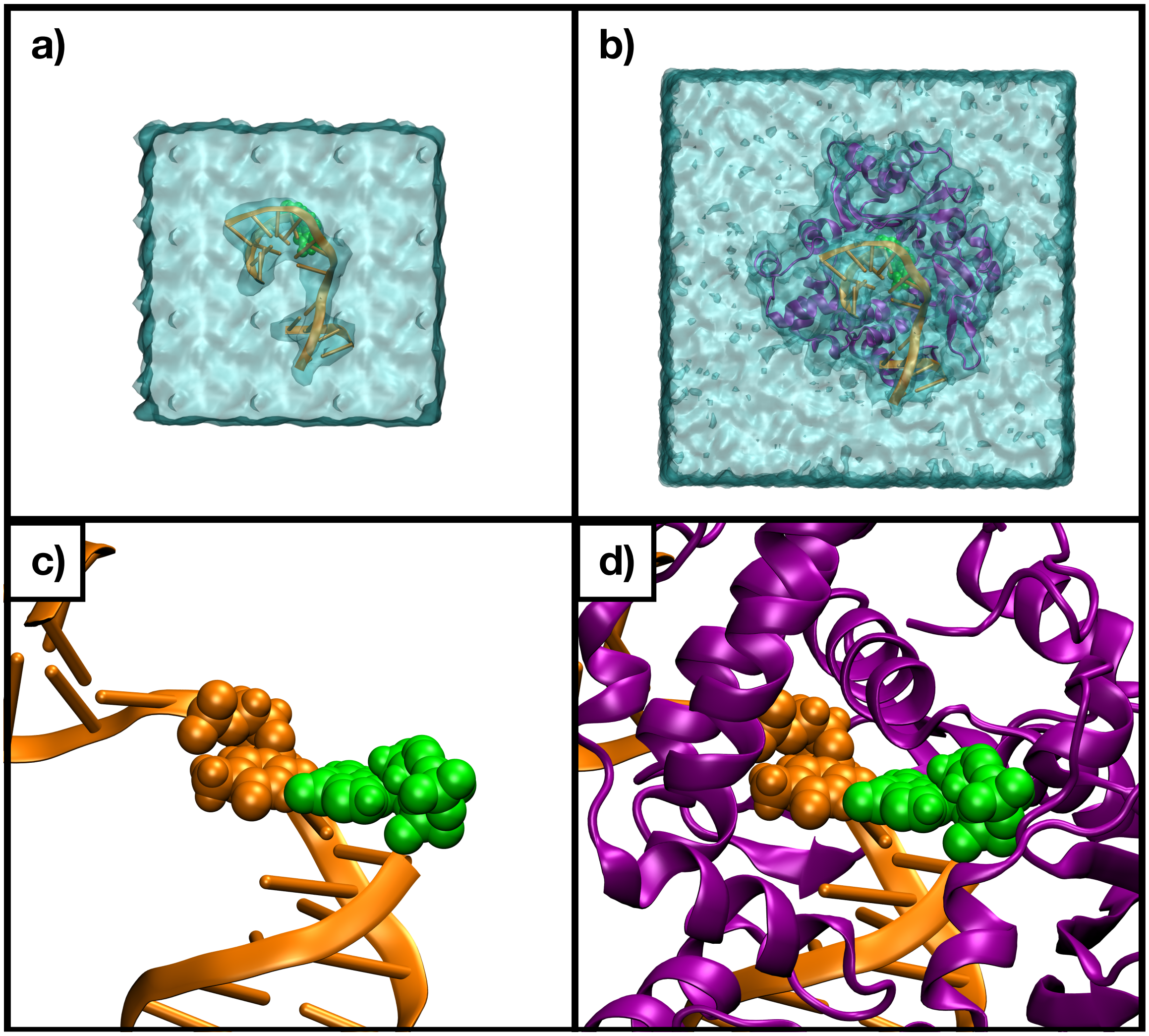

Figure 2: The simulation systems for classical MD and hybrid QM/MM MD. Panel a) is the enzyme-less solvated DNA (orange cartoon) system with GTP (green space-filled) bound in the wobble(G-T) configuration, and shown in detail in panel c). Panel b) shows the enzyme-DNA complex of Polymerase-λ (purple cartoon) with the same DNA and GTP representations as panel a). Panel d) shows the wobble(G-T) configuration in the thumb domain of the enzyme.

$$\Delta = \sqrt{(a - a_{\mathrm{TRS}})^2 + (b - b_{\mathrm{TRS}})^2 + (c - c_{\mathrm{TRS}})^2} \quad (1)$$

- To facilitate the new QM/MM calculations, we updated the author list.

- We updated the abstract to reflect the new QM/MM calculations.
The misincorporation of a non-complimentary DNA base in the polymerase active site is a critical source of replication errors that can lead to genetic mutations. In this work, we model the mechanism of wobble mispairing and the subsequent rate of misincorporation errors by coupling first-principles quantum chemistry calculations to an open quantum systems master equation. This methodology allows us to accurately calculate the proton transfer between bases, allowing the misincorporation and formation of mutagenic tautomeric forms of DNA bases. Our calculated rates of genetic error formation are in excellent agreement with experimental observations in DNA. Furthermore, our quantum mechanics/molecular mechanics model predicts the existence of a short-lived "tunnelling-ready" configuration along the wobble reaction pathway in the polymerase active site, dramatically increasing the rate of proton transfer by a hundredfold, demonstrating that quantum tunnelling plays a critical role in determining the transcription error frequency of the polymerase.

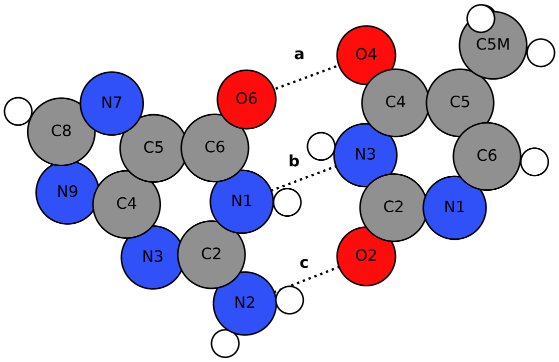

Figure 3: Reaction coordinate definition for the statistical sampling of the tunnelling ready state. a,b, and c are the three reaction coordinates used to quantify the compression of the G-T wobble dimer.

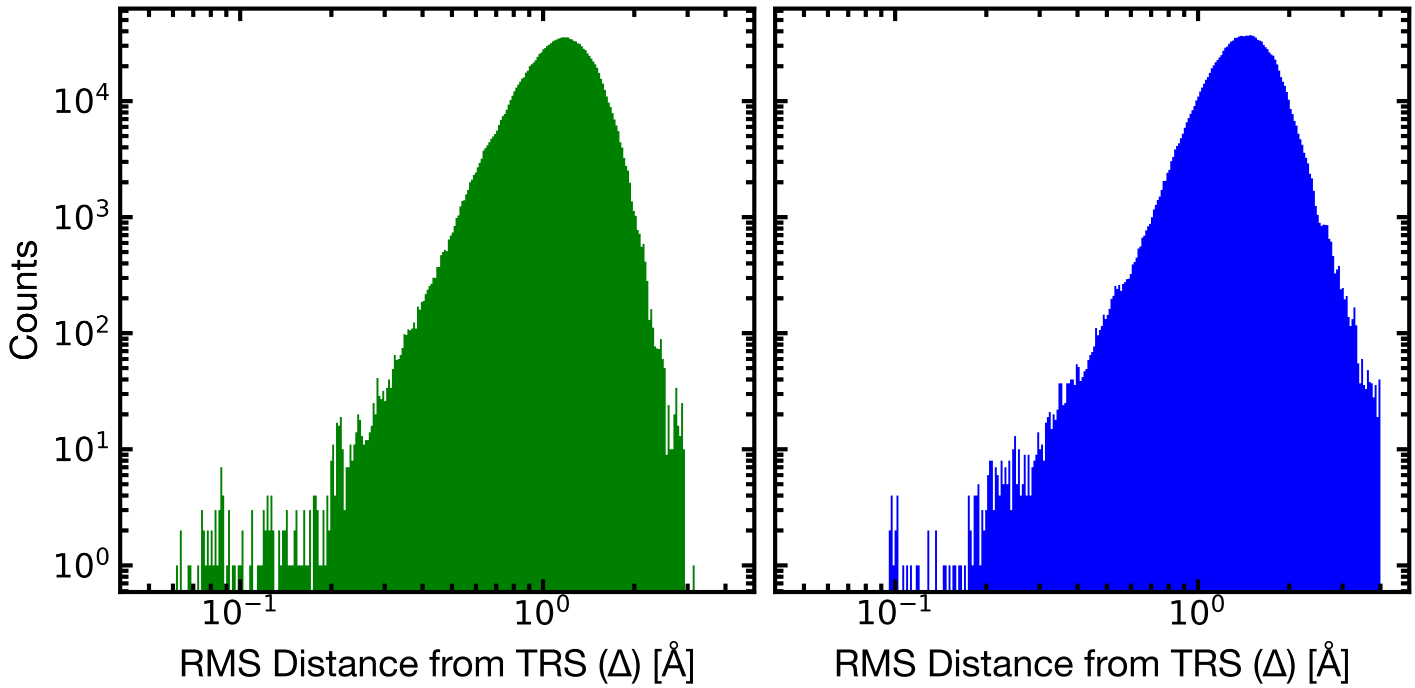

Figure 4: Histogram of the compression metric (Δ) for the wobble(G-T) dimer in Polymerase (left panel) and just with DNA (right panel). Each panel corresponds to data aggregated from over 180 ps of ensemble QM/MM MD.

1.2 Point 2 - Lack of comparison with competing sources of mutation

The reviewer raises the issue that the paper does not compare the wobble mismatch to other competing sources of mutation.

> *2. This is a too narrow view for a complex phenomenon. Authors must compare these values with other proton transfer equilibria in competition during mutation.*

Response We thank the reviewer for the suggestion. We agree that adding this comparison could aid the analysis and highlight the results to extract real biological conclusions.

- To address this issue, we highlight the mechanism for proton transfer before the helicase enzyme induces strand separation. The changes can be found in the discussion and conclusions on page 12.

  In summary, we have employed quantum chemical calculations to determine the reaction pathway of several reactions for generating tautomers of the G-T wobble mispair. We applied an open quantum systems approach to account for the decoherent and dissipative local environment [18] and identified quantum and classical contributions to the reaction rates. For the wobble(G-T) $\rightleftharpoons$ G*-T mechanism, we find that the reaction proceeds via a step-wise process involving G-T*. Consequently, we focused on wobble(G-T) $\rightleftharpoons$ G-T*. The proton transfer reaction from the wobble to the Watson-Crick pathway has a significantly high and broad reaction barrier, which implies an insignificant contribution from quantum tunnelling and a slow classical rate. We noted that for the wobble(G-T)$\rightleftharpoons$G-T* reaction to occur, the nucleotide dimer must first be compressed into a "tunnelling-ready" state, we probed this state using QM/MM MD to determine how likely it is populated in a biologically relevant thermal ensemble. We evaluated that this state is more likely to be populated in the polymerase environment and leads to an increase in quantum tunnelling.

  As highlighted by previous computational studies, the role of proton transfer in spontaneous mutation is a complex affair [8, 11, 19]. However, the proton transfer mechanism in the polymerase is a prominent candidate as a source of mutations as it is later in the replication cycle and could play a more significant role than other equilibria competing during mutation. Furthermore, for mechanisms involving double-stranded Watson-Crick DNA, it needs to be clarified if the helicase or another mechanism reduces the proton transfer populations via electrostatic destabilisation or exonuclease proofreading mechanisms.

  To conclude, our model predicts tunnelling rates that match the experimental NMR observed rates to a high degree of accuracy—opening the possibility that quantum mechanics is required to explain the biologically relevant functionality of polymerase.

- We further expanded the discussion on the other proton transfer mechanism. The changes can be found in the introduction on page 3.

  As previously demonstrated, once the tautomer is formed and the DNA is opened, it is stabilised and is unlikely to revert to its canonical form due to a prohibitively large reaction barrier [1, 20]. However, it still needs to be determined to what degree environmental effects play a role in destabilising the tautomer. Some initial evidence suggests that the DNA [19] environment reduces the reverse barrier, but it is unclear for the DNA and helicase complex.

1.3 Point 3 - The chemical system allows for unrealistic conformation

The reviewer raises the issue that the choice of the chemical system allows for unrealistic conformation due to the lack of the polymerase active site in the calculations.

The selected chemical system is also a major drawback. As correctly stated by the authors, the polymerase active site is a critical source of replication errors that might lead to genetic mutations. In other words, these mispairs appear at the polymerase active site, but authors used an isolated base pair. The impact of the pockets is completely missing, so that numeric values cannot be extrapolated nor compared to real conditions.

The reviewer then summarises with the following:

3. The chemical system allows for unrealistic conformation. These mutations appear in the polymerase binding site. The model based on an isolated bases pairs must be improved. Biological media might also impact.

Response We thank the reviewer for drawing our attention to this point. Our results, specifically the tunnelling corrected reaction rates, agree quantitatively with recent NMR [3–5], and computational studies [8], which suggest that the population of mispairs depends on the environment, such as the local nucleic acid sequence, solvation, and complex polymerase active site. In light of the reviewer's suggestion, we have made significant changes to our manuscript.

- We revised our manuscript to signpost this issue. The changes can be found on page 4.
  ... The free energy contributions reduce the forward barrier by 20% and the reverse barrier by 30%, resulting in a free energy profile consistent with Li *et al.* [8]. A summary can be found in supplementary note 1, and a detailed comparison of the reaction barrier parameters to the literature in note 3.
- We revised the discussion and analysis of the rates. The changes can be found on pages 7 and 8.
  Firstly, we determine the quantum and classical rates for reaction 1 using our open quantum systems approach. Reaction 1 has a prohibitively high and wide reaction barrier (see Fig. 2), resulting in a low classical and quantum reaction rate. We evaluate that the quantum-to-classical ratio is small, $\kappa = 1.02$, suggesting that tunnelling is negligible; here, in this case, dissipative and decoherent effects from the biological environment suppress the tunnelling. We find that the overall reaction rate is dominated by an over-the-barrier classical mechanism, with a value of $5.244 \times 10^{-1}\,\mathrm{s}^{-1}$ - which is consistent with both the experimental value (0.6-68 s^{-1} [3,5]) of the G-T wobble system in DNA. The reaction rate is several orders of magnitude smaller than the dNTP unbinding rate, which is of the order 70 000 s^{-1} [3]. Furthermore, we determine the effect of isotopic substitutions on the reaction rate and found that the reaction rate is essentially unaffected by deuterium substitution (KIE=1.1). Consequently, due to the slow reaction rate, the dNTPs unbinding rate and subsequent base rejection compete with the proton transfer mechanism. As a result, statistically, some of the wobble mismatches will eventually diffuse from the polymerase's active site before proton transfer occurs. Since the diffusion timescale competes with the proton transfer timescale, the final population of tautomers incorporated will be reduced as accounted for by the kinetic network in Ref. [3].
- In addition, we have added further quantum corrected rates for the wobble to Watson-Crick and the Watson-Crick to Watson-Crick reaction profiles. To do this, we adopt the minimum free energy paths for the tautomerisation reaction from Li *et al.* and apply and compare how the quantum bath affects the tunnelling, thus providing a direct comparison of the environmental impact of the mispairing mechanism. The changes to the main document are on pages 8.
  To compare how the change of environment and subsequent change to the reaction profile impacts the tunnelling, we extract the free energy pathway data from Li *et al.* [8] and apply our tunnelling approach. A detailed description can be found in supplementary note 3. In summary, we determined that regardless if the G-T wobble is exclusively in an aqueous solution or a more complex DNA environment, the tunnelling is primarily suppressed to the degree that it is insignificant.
- To facilitate the above changes, we added a new section to the supplementary information file. See supplementary note 3 on pages 15–17.
  To compare how the environment has an impact on the tunnelling, we extract the free energy pathway data from Li *et al.* [8] using WebPlotDigitizer - a web-based tool to extract numerical data from plots [21]. We then have to scale the free energy pathway so that the energy is a function of the reaction path instead of the image index. To do this, we assume that each reaction follows the same path as the ML-NEB data we report. Consequently, we perform a linear rescaling to map the free energy pathway onto our data.

  Next, using the extracted free energy pathway, which is now a function of the reaction path, we perform a constrained least-squares fit to adopt the reaction profile into the open quantum systems Hamiltonian. See supplementary note 1 for further details. The result of the fit is summarised in table 1.
- We compare the potential energy profiles to the polymerase and DNA complex given by Li and provide an analysis of the difference, see supplementary note 3 on pages 15–17.

Table 1: Summary of the potential parameters used to describe the proton transfer reactions. The following parameters are defined: ω_0 spring constant of the barrier, L_0 is the displacement, q_0 is the additional tilt parameter, and ΔE well energy.

Parameter	wob(G-T) $\rightleftharpoons$ G-T*			G*-T $\rightleftharpoons$ G-T*		
	Aqueous	B-DNA	Poly-λ	Aqueous	B-DNA	Poly-λ
ω_0	0.001 41 AUT	0.001 41 AUT	0.001 41 AUT	0.001 41 AUT	0.001 41 AUT	0.001 41 AUT
L_0	12.76 a_0	12.76 a_0	12.76 a_0	12.76 a_0	12.76 a_0	12.76 a_0
q_0	12.76 a_0	12.76 a_0	12.76 a_0	12.76 a_0	12.76 a_0	12.76 a_0
ΔE	$-0.005\,56\,E_h$	$-0.005\,56\,E_h$	$-0.005\,56\,E_h$	$-0.005\,56\,E_h$	$-0.005\,56\,E_h$	$-0.005\,56\,E_h$

Table 2: Summary of the quantum and classical contributions to the reactions. With terms, forward reaction rate k_f, reverse reaction barrier k_r, reactant lifetime τ_f, product lifetime τ_r, chemical equilibrium value K_{eq}, quantum vs classical rate contribution κ, KIE (kinetic isotope effect).

Parameter	wob(G-T) $\rightleftharpoons$ G-T*			G*-T $\rightleftharpoons$ G-T*		
	Aqueous	B-DNA	Poly-λ	Aqueous	B-DNA	Poly-λ
k_f	$1.775 \times 10^{-1}\,s^{-1}$	$8.911\,s^{-1}$	$3.212 \times 10^{-6}\,s^{-1}$	$2.493 \times 10^{8}\,s^{-1}$	$5.959 \times 10^{8}\,s^{-1}$	$7.494 \times 10^{7}\,s^{-1}$
k_r	$2.274 \times 10^{10}\,s^{-1}$	$6.204 \times 10^{5}\,s^{-1}$	$1.612 \times 10^{-6}\,s^{-1}$	$2.265 \times 10^{8}\,s^{-1}$	$7.568 \times 10^{8}\,s^{-1}$	$4.215 \times 10^{9}\,s^{-1}$
τ_f	$5.635\,s^{-1}$	$1.122 \times 10^{-1}\,s^{-1}$	$3.113 \times 10^{5}\,s^{-1}$	$4.010 \times 10^{-9}\,s^{-1}$	$1.678 \times 10^{-9}\,s^{-1}$	$1.334 \times 10^{-8}\,s^{-1}$
τ_r	$4.397 \times 10^{-11}\,s^{-1}$	$1.612 \times 10^{-6}\,s^{-1}$	$6.205 \times 10^{5}\,s^{-1}$	$4.415 \times 10^{-9}\,s^{-1}$	$1.321 \times 10^{-9}\,s^{-1}$	$2.373 \times 10^{-10}\,s^{-1}$
K_{eq}	7.804×10^{-12}	1.436×10^{-5}	1.993	1.101	7.874×10^{-1}	1.778×10^{-2}
κ	1.01	1.01	1.03	3.85	2.41	4.84
KIE	1.0	1.0	1.0	2.19	1.67	2.51

Fig. 5 compares our ML-NEB data of the wobble(G-T)$\rightleftharpoons$G-T* (panel a) and G*-T$\rightleftharpoons$G-T* (panel b) reaction with the free energy curve from Li *et al.* [8].

For panel a), the wobble reaction, all reaction paths have a similar initial energy trend, corresponding to the classical sliding and compression of the G-T wobble to facilitate the proton transfer; this mechanism is described in Fig. 2 of the main document. Furthermore, there is little to no variation between the environmental systems suggesting that the initial path is similar irrespective of the local environment. However, our barrier is slightly smaller than the one of the DNA/polymerase system but larger than the isolated B-DNA and DNA/aqueous solution barriers. While our free energy corrected barrier shown in table 2 is within 14% of the B-DNA. Similarly, the ML-NEB reaction energy matches the B-DNA system within 16%. However, the barrier is significantly higher for the polymerase system, but the reaction energy is much lower. On the other hand, for Fig. 5b), in the Watson-Crick to Watson-Crick reaction, our energy is larger than the free energy profiles. However, with our free energy profiles, our barrier is significantly reduced. Consequently, overall our energy profiles are within reasonable agreement with Li *et al.* [8] B-DNA system.

- We compare the environmental effects on the quantum tunnelling to the polymerase and DNA complex given by Li. See supplementary note 3 on pages 15–17.

Finally, we use the extracted free energy potentials to determine the rates due to classical over-the-barrier hopping and tunnelling. The results are summarised in table 2. Here, we explore the classical and quantum rates and all the previously calculated parameters.

Due to the wide barrier, we find an insignificant amount of tunnelling for the wobble mechanism for all environments. This finding is consistent with our previous finding regarding our ML-NEB potential. Furthermore, as there is little tunnelling, the KIE is also low, again indicating that the reaction is isotopic independent and predominantly classical. Thus if we adopt the Li *et al.* models and ignore the frozen approximation, as we detailed in supplementary note 1, we conclude that there is little dependence on the choice of environments on the tunnelling and instead, the proton transfer is an over-the-barrier classical behaviour. Here we note that the classical B-DNA rate is consistent with the NMR data [3–5].

On the other hand, the reaction weakly depends on the local environment for the Watson-Crick to Watson-Crick reaction, varying from 2.41 to 4.84. Overall, the quantum-to-classical ratio increases in the polymerase compared to the aqueous system due to the classical rate dropping quicker than the quantum rate, as the polymerase system has a higher barrier but a similar width.

- We added further quantum corrected rates for the wobble to Watson-Crick and the Watson-Crick to Watson-

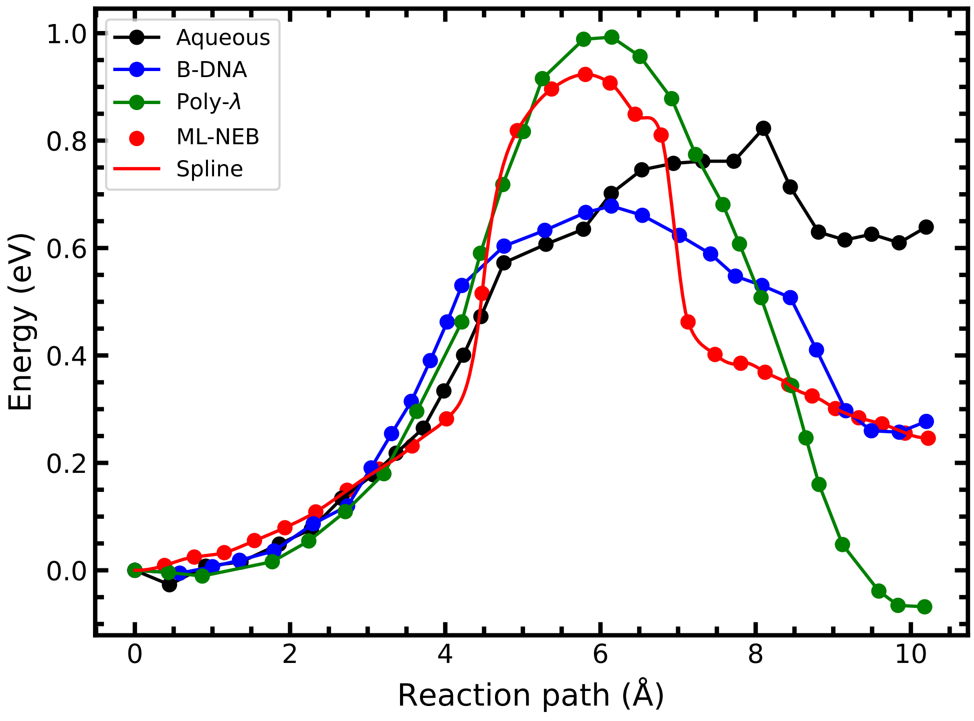

(a) wobble(G-T)⇌G-T*

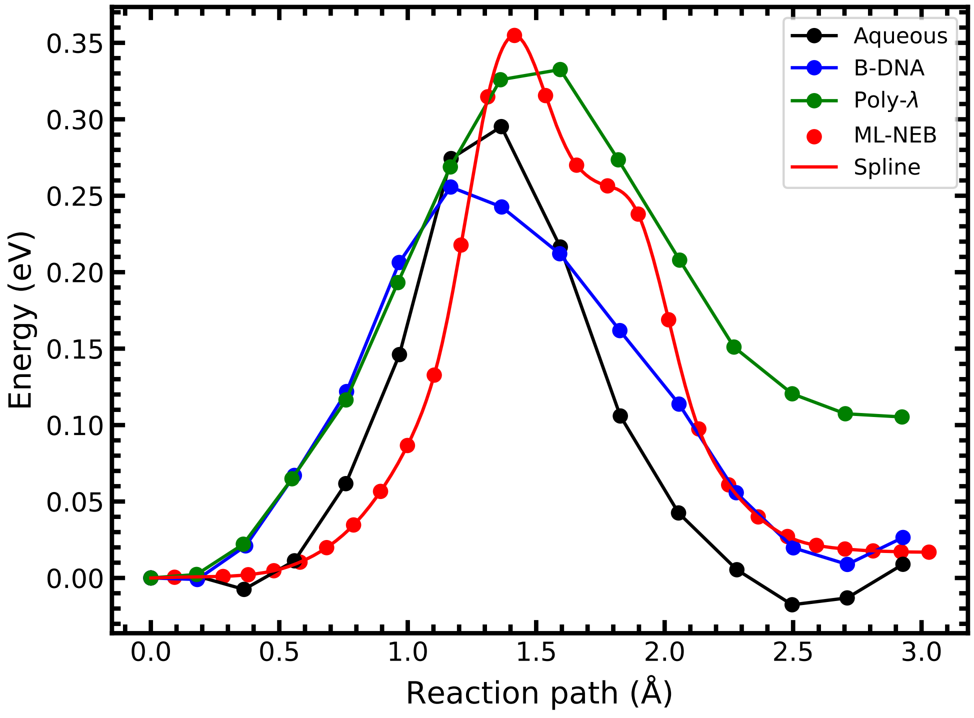

(b) G*-T⇌G-T*

Figure 5: Comparison of the Minimum energy paths of wobble(G-T)⇌G-T* and G*-T⇌G-T* reactions. Here, the aqueous, B-DNA, and poly-λ are taken from Li *et al.* [8]. In red is the data obtained using a machine-learning approach to the nudged elastic band method.

Crick reaction profiles. To do this, we adopt the minimum free energy paths for the tautomerisation reaction from Li *et al.* and apply and compare how the quantum bath affects the tunnelling. Thus, consequently providing a direct comparison of the environmental impact of the mispairing mechanism. The changes to

the main document are on pages 8.

To compare how the change of environment and subsequent change to the reaction profile impacts the tunnelling, we extract the free energy pathway data from Li *et al.* [8] and apply our tunnelling approach. A detailed description can be found in supplementary note 3. In summary, we determined that regardless if the G-T wobble is exclusively in an aqueous solution or a more complex DNA environment, the tunnelling is primarily suppressed to the degree that it is insignificant.

2 Referee 2

Recommendation: This paper is publishable subject to minor revisions noted. Further review is not needed.

This manuscript describes a way to determine the reaction pathway of several reactions for generating tautomers of the G-T wobble mispair. The authors account for the bases' decoherent and dissipative local environment and identified quantum and classical contributions to the reaction rates by an open quantum systems approach. And the authors found that the reaction for the wobble(G-T) ↔ G-T proceeds via a stepwise process. I thought it was a very interesting result.*

1) p6 The reader would like to know how much the fifth and sixth terms on the right side of Equation 1, which represent the interaction between DNA and its environment, affected the reaction in the results of this calculation. This would show that the environment surrounding the DNA plays an important role in the wobble(G-T) ↔ G-T reaction. Please explain the ratio of contributions of the Schrödinger dynamics term, the dissipation term, and the decoherence term, respectively.*

2) SUPPLEMENTARY NOTE 2 The authors state in SUPPLEMENTARY NOTE 2 that dissipation and decoherence are derived from the coupling to the quantum bath. Where is this quantum bath derived from in DNA? I think this explanation will convince biophysicists.

Response We thank Referee 2 for their interest and for contributing their valuable perspective on the core concepts covered in the manuscript.

2.1 Point 1 - Terms corresponding to the quantum system interacting with the bath need clarifying

The reader would like to know how much the fifth and sixth terms on the right side of Equation 1, which represent the interaction between DNA and its environment, affected the reaction in the results of this calculation. This would show that the environment surrounding the DNA plays an important role in the wobble(G-T) ↔ G-T reaction. Please explain the ratio of contributions of the Schrödinger dynamics term, the dissipation term, and the decoherence term, respectively.*

Response We thank the referee for raising this excellent point. The referee suggested that the idea behind open quantum systems needs to be appropriately introduced. We do indeed need to clarify the impact of the bath further.

- Following the referees' comment, we have added more discussion on the terms to pages 6 and 7.
  The advantage of employing an open quantum system model is that it incorporates the interactions with the local environment in the quantum dynamics. These interactions significantly affect the system's dynamics and can either impede or encourage the system's evolution, known as a quantum Zeno or anti-Zeno effect [22]. Furthermore, the coupling to the environment results in quantum dissipation, such that the information in the system is lost to its environment and decoherence, where a quantum system loses its wave-like properties. As a consequence, classical behaviour emerges.

2.2 Point 2 - The origin of the bath is not clear

The authors state in SUPPLEMENTARY NOTE 2 that dissipation and decoherence are derived from the coupling to the quantum bath. Where is this quantum bath derived from in DNA? I think this explanation will convince biophysicists.

Response Thanks for raising the issue that this broader description is missing.

- We have provided further explanation of the derivation of the bath's properties in the supplementary information on page 10.
  An ideally isolated quantum system, particularly in biology, is unlikely. Instead, the environment is constantly interacting with the system. In the cellular environment, there is a constant energy flow between the system and the environment through vibrations and collisions with the surrounding solvent and proteins, constantly perturbing the quantum system. Once decoherence sets in, we might expect entirely classical behaviour to emerge. To describe this transition region, we require a theoretical framework to describe the protons in DNA using an open quantum systems approach.

  The idea of an open quantum system is to incorporate interactions with the local environment. These interactions significantly change the system's dynamics and result in quantum dissipation and decoherence.

The general idea is to couple a system Hamiltonian $\hat{H}_{\mathrm{S}}$ with a bath $\hat{H}_{\mathrm{B}}$ via an interaction $\hat{H}_{\mathrm{I}}$,

$$\hat{H}_{\mathrm{SB}} = \hat{H}_{\mathrm{S}} + \hat{H}_{\mathrm{B}} + \hat{H}_{\mathrm{I}}. \quad (2)$$

Here the interaction term generates quantum and classical correlations between the system and the environment [23].

3 Further corrections

This section covers additional minor corrections that the referees have not directly suggested. However, we believe they will improve the general quality of the paper. Please see the changes below.

3.1 Further correction 1

We amended the acknowledgements.

This work was made possible through the support of the Leverhulme Trust doctoral training centre grant number DS-2017-079 and from the John Templeton Foundation grant number 62210. M.S. is grateful for support from the Royal Society (URF/R/191029). We acknowledge helpful discussions with the members of the Leverhulme Quantum Biology Doctoral Training Centre; particular thanks go to Johnjoe McFadden. Further thanks go to Antonio Pantelias, who offered many productive conversations. In addition, the authors thank the University of Surrey for access to Eureka. Via our membership of the UK's HEC Materials Chemistry Consortium, funded by EPSRC (EP/R029431) and the UKCP Consortium, funded by EPSRC grant ref EP/P022561/1, this work used the ARCHER2 UK National Supercomputing Service.

3.2 Further correction 2

We replaced all instances of Pengfei *et al.* with Li *et al.* in the supplementary file.

3.3 Further correction 3

To increase readability, we removed the Open Quantum System (OQS) acronym from both the manuscript and the supplementary file.

3.4 Further correction 4

We updated the description and analysis of Fig. 3; the changes can be found on page X.

Here we explore the minimum energy pathway of proton transfer in the tunnelling-ready state. Further details of the methods can be found in supplementary note 1. The subsequent minimum energy pathway is shown in Fig. 3. Here, the reaction pathway shows three pseudo-minima corresponding to the bases already part-way slid into a Watson-Crick-like shape, the second, where the proton has transferred to the other base, and the third, the return of the proton back to the same base. The last two minima indicate that if we assume that the proton transfer is much faster than the rest of the atomic motion during the reaction, a bifurcation of the reaction pathway is possible. In fact, after the first initial proton transfer, the rest of the atoms could rearrange, trapping the population in the middle well.

3.5 Further correction 5

We fixed minor grammar and spacing issues in both the article and supplementary files.

References

[1] L Slocombe, JS Al-Khalili, and M Sacchi. Quantum and classical effects in dna point mutations: Watson–crick tautomerism in at and gc base pairs. *Physical Chemistry Chemical Physics*, 23(7):4141–4150, 2021.

[2] Louie Slocombe, Marco Sacchi, and Jim Al-Khalili. An open quantum systems approach to proton tunnelling in dna. *Communications Physics*, 5(1):1–9, 2022.

[3] Isaac J Kimsey, Eric S Szymanski, Walter J Zahurancik, Anisha Shakya, Yi Xue, Chia-Chieh Chu, Bharathwaj Sathyamoorthy, Zucai Suo, and Hashim M Al-Hashimi. Dynamic basis for dg• dt misincorporation via tautomerization and ionization. *Nature*, 554(7691):195–201, 2018.

[4] Isaac J Kimsey, Katja Petzold, Bharathwaj Sathyamoorthy, Zachary W Stein, and Hashim M Al-Hashimi. Visualizing transient watson–crick-like mispairs in dna and rna duplexes. *Nature*, 519(7543):315–320, 2015.

[5] Atul Rangadurai, Eric S Szymanski, Isaac Kimsey, Honglue Shi, and Hashim M Al-Hashimi. Probing conformational transitions towards mutagenic watson–crick-like g· t mismatches using off-resonance sugar carbon r 1ρ relaxation dispersion. *Journal of Biomolecular NMR*, pages 1–15, 2020.

[6] Weina Wang, Homme W Hellinga, and Lorena S Beese. Structural evidence for the rare tautomer hypothesis of spontaneous mutagenesis. *Proceedings of the National Academy of Sciences*, 108(43):17644–17648, 2011.

[7] Alexey Rozov, Natalia Demeshkina, Eric Westhof, Marat Yusupov, and Gulnara Yusupova. Structural insights into the translational infidelity mechanism. *Nature communications*, 6(1):1–9, 2015.

[8] Pengfei Li, Atul Rangadurai, Hashim M. Al-Hashimi, and Sharon Hammes-Schiffer. Environmental effects on guanine-thymine mispair tautomerization explored with quantum mechanical/molecular mechanical free energy simulations. *Journal of the American Chemical Society*, 142(25):11183–11191, 2020. PMID: 32459476.

[9] Ol'ha O. Brovarets and Dmytro M. Hovorun. Quantum dancing of the wobble g•t(u/5bru) nucleobase pairs and its biological roles. *Chemical Physics Impact*, 1:100006, 2020.

[10] Kei Odai and Keisho Umesaki. Kinetic study of transition mutations from g–c to a–t base pairs in watson–crick dna base pairs: Double proton transfers. *The Journal of Physical Chemistry A*, 125(37):8196–8204, 2021.

[11] Shreya Chandorkar, Shampa Raghunathan, Tanashree Jaganade, and U Deva Priyakumar. Multiscale modeling of wobble to watson–crick-like guanine–uracil tautomerization pathways in rna. *International journal of molecular sciences*, 22(11):5411, 2021.

[12] Henk Bekker, HJC Berendsen, EJ Dijkstra, S Achterop, R Vondrumen, David Vanderspoel, A Sijbers, H Keegstra, and MKR Renardus. Gromacs-a parallel computer for molecular-dynamics simulations. In *4th International Conference on Computational Physics (PC 92)*, pages 252–256. World Scientific Publishing, 1993.

[13] Katarzyna Bebenek, Lars C Pedersen, and Thomas A Kunkel. Replication infidelity via a mismatch with watson–crick geometry. *Proceedings of the National Academy of Sciences*, 108(5):1862–1867, 2011.

[14] Katarina Hart, Nicolas Foloppe, Christopher M Baker, Elizabeth J Denning, Lennart Nilsson, and Alexander D MacKerell Jr. Optimization of the charmm additive force field for dna: Improved treatment of the bi/bii conformational equilibrium. *Journal of chemical theory and computation*, 8(1):348–362, 2012.

[15] Robert B Best, Xiao Zhu, Jihyun Shim, Pedro EM Lopes, Jeetain Mittal, Michael Feig, and Alexander D MacKerell Jr. Optimization of the additive charmm all-atom protein force field targeting improved sampling of the backbone ϕ, ψ and side-chain $\chi1$ and $\chi2$ dihedral angles. *Journal of chemical theory and computation*, 8(9):3257–3273, 2012.

[16] HJC Berendsen, JR Grigera, and TP Straatsma. The missing term in effective pair potentials. *Journal of Physical Chemistry*, 91(24):6269–6271, 1987.

[17] Thomas D Kühne, Marcella Iannuzzi, Mauro Del Ben, Vladimir V Rybkin, Patrick Seewald, Frederick Stein, Teodoro Laino, Rustam Z Khaliullin, Ole Schütt, Florian Schiffmann, et al. Cp2k: An electronic structure and molecular dynamics software package-quickstep: Efficient and accurate electronic structure calculations. *The Journal of Chemical Physics*, 152(19):194103, 2020.

[18] Amir O Caldeira and Anthony J Leggett. Path integral approach to quantum brownian motion. *Physica A: Statistical mechanics and its Applications*, 121(3):587–616, 1983.

[19] A Gheorghiu, PV Coveney, and AA Arabi. The influence of base pair tautomerism on single point mutations in aqueous dna. *Interface focus*, 10(6):20190120, 2020.

[20] Louie Slocombe, Max Winokan, Jim Al-Khalili, and Marco Sacchi. Proton transfer during dna strand separation as a source of mutagenic guanine-cytosine tautomers. *Communications Chemistry*, 5(1):144, Nov 2022.

[21] Ankit Rohatgi. Webplotdigitizer: Version 4.6, 2022.

[22] Zixian Zhou, Zhiguo Lü, Hang Zheng, and Hsi-Sheng Goan. Quantum zeno and anti-zeno effects in open quantum systems. *Physical Review A*, 96(3), 2017.

[23] Heinz-Peter Breuer and Francesco Petruccione. *The Theory of Open Quantum Systems*. Oxford University Press on Demand, 2007.
